# Evolutionary selection of trimethoprim-resistant *dfrA* genes in lytic phages affects phage and host fitness during infection

**DOI:** 10.1126/sciadv.adt4817

**Published:** 2025-09-26

**Authors:** Kai Wang, Jikai Xu, Xudong Li, Pengfei Zhu, Ruijie Suo, Xiaowei Lu, Haixin Luo, Li Chen, Rong Wen, Chengbo Zheng, Alejandra Bravo, Mario Soberón, Jinshui Zheng, Ming Sun, Donghai Peng

**Affiliations:** ^1^ National Key Laboratory of Agricultural Microbiology, Hubei Hongshan Laboratory, Huazhong Agricultural University, Wuhan 430070, Hubei, China.; ^2^ College of Life Science and Technology, Huazhong Agricultural University, Wuhan 430070, China.; ^3^ College of Informatics, Huazhong Agricultural University, Wuhan, China.; ^4^ Instituto de Biotecnología, Universidad Nacional Autónoma de México, Apdo. postal 510-3, Cuernavaca 62250 Morelos, Mexico.

## Abstract

Temperate phages and prophages are well-known carriers of antibiotic resistance genes (ARGs) facilitating their transmission. In contrast, lytic phages rarely harbor functional ARGs. However, by a lenient threshold search strategy combined with a machine learning approach based on structural similarity, here we identified 9419 potential ARGs within lytic phages. We showed that potential trimethoprim-resistance dihydrofolate reductase (*dfrA*) genes enriched in lytic phages could confer trimethoprim resistance to *Escherichia coli*. Sequence analysis revealed that lytic phages rarely transfer these potential *dfrA* genes into their bacterial hosts. Functional studies showed that these *dfrA* genes not only enhance phage reproduction in the presence of trimethoprim but also promote bacterial growth during phage infection. These results highlight that abundant functional ARGs were selected in evolution to improve lytic phage reproduction by promoting bacterial growth during infection, suggesting that *dfrA* genes play important roles in evolutionary mutualism between lytic phages and their hosts.

## Teaser

Lytic phages for phage therapy carry abundant ARGs that rarely spread to bacteria but enhance phage and host fitness.

## INTRODUCTION

Antibiotic resistance of bacterial pathogens poses a global threat on public health (*1*, *2*). It was estimated that by 2050, antibiotic resistance could cause up to 10 million deaths per year and more than $100 trillion USD in global losses (*3*). The main reason for the emergence of bacterial antibiotic resistance is their acquisition of antibiotic resistance genes (ARGs). ARGs cause antibiotic resistance through different mechanisms, such as inactivating antibiotics, protecting antibiotic cellular targets, and reducing intracellular antibiotic concentration either by efflux pumps or changing cell membrane permeability (*4*). Therefore, finding ARGs and reducing their spread among different organisms is urgently needed.

Plasmids are the primary reservoirs of ARGs (*5*). Furthermore, plasmid-mediated horizontal gene transfer (HGT) represents the main driving force for spreading ARGs (*6*). However, phages could also mediate HGT playing an increasingly important role in ARGs spreading by transduction (*7*–*9*). Analysis of prophage genomes has revealed that certain prophages can carry multiple ARGs. A unique and prevalent group of such prophages in *Streptococcus*, known as SMphages, exhibits a wide host range; shares high-sequence identity with Φm46.1, Φ10394.4, λSa04, and ΦSsUD.1; and encodes up to 25 ARG subtypes (*10*). In addition, it has been shown that 4.2% (60 of 1416) of phage-plasmids encode up to 184 ARG subtypes (*11*). Furthermore, prophages have extensively facilitated the dissemination of ARGs among different bacteria through lysogenic conversion or conjugative transfer (*10*, *11*). However, large-scale analysis of phage genomes revealed that 1% (43 of 4258) of temperate phages harbor reported ARGs (*12*), while lytic phages lack reported ARGs (*12*–*14*). Also, analysis of the abundance of ARG sequences in environmental metagenome viromes showed that no ARGs were detected in the virome contigs analyzed using a conservative threshold (identity ≥80% and query coverage ≥85%) (*15*). Unexpectedly, if lenient thresholds (*E*-value thresholds ≤1 × 10^−5^ and query coverage ≧ 60% for BLASTp, or *E*-value thresholds ≤1 × 10^−5^ and a score ≧ 40 for HMMSCAN) were used, it was shown that 0.47% (25 of 5295) of the analyzed contigs carried potential ARGs, including two functional β-lactamases (*15*). Therefore, it is necessary to comprehensively review the diversity and distribution of ARGs in lytic phages as well as to evaluate the risk of lytic phage–borne ARGs dissemination among bacteria.

In this study, we undertook lenient threshold search strategies and pairwise comparative modeling (PCM) based on machine learning of structural similarity (*16*), leading to the identification of 11,665 potential ARGs across 26,697 phage genomes, where 80.74% were carried by lytic phages. Further analysis of ARGs among lytic phages revealed that functional trimethoprim-resistance dihydrofolate reductase (*dfrA*) genes were enriched and widely distributed among multiple lytic phages. Nevertheless, our findings also revealed that lytic phages rarely spread these ARGs to bacteria. In addition, we show that lytic phage–borne *dfrA* genes enhance phage fitness but also regulate host behavior, suggesting that ARGs play important roles in evolutionary mutualism between lytic phages and their hosts.

## RESULTS

### Phages carry abundant potential ARGs

**Fig. 1.**
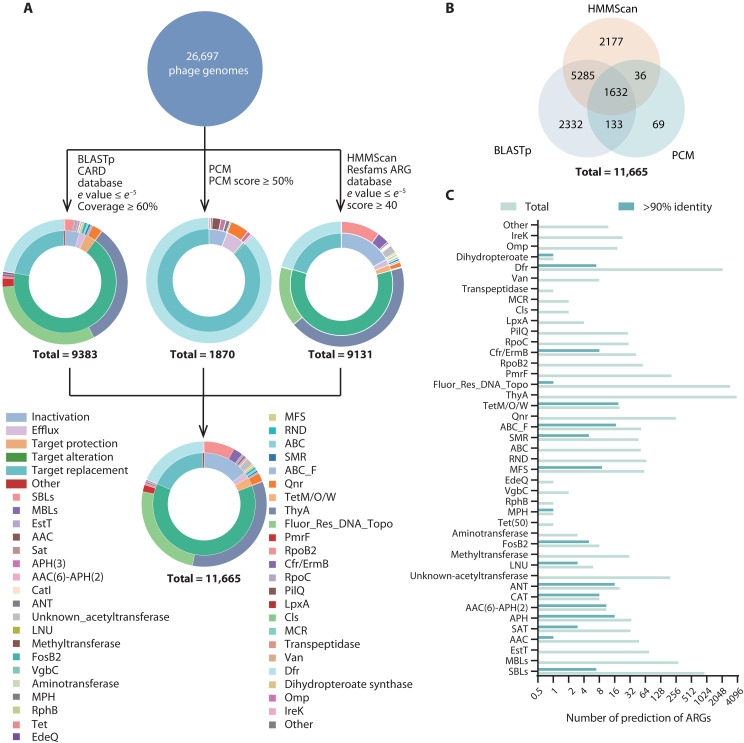
Identification and analysis of potential ARGs in all phage isolates. (**A**) Strategies used to search potential ARGs in all phage isolates and identification of ARGs profiles by BLASTp analysis, machine-learning PCM methods, and HMMScan. All identified potential ARGs were functionally annotated and classified. The results are visualized in a sunburst chart, with the inner layer classifying ARGs by their resistance mechanism and the outer layer providing functional annotation of individual ARGs. (**B**) Venn diagram of potential ARGs identified. Data show that 885 ARG candidates were identified by the three methods. (**C**) Number of different types of potential ARGs identified and those that exhibit more than 90% identity with reported ARGs.

To evaluate the impact of different identity thresholds on the detection of potential ARGs encoded by phage genomes available in National Center for Biotechnology Information (NCBI), we performed a sensitivity analysis using identity cutoffs ranging from 30 to 90% (table S1). The analysis revealed that using identity thresholds ≥50% drastically reduced the number of detected potential ARGs, particularly in lytic phages, where only 125 potential ARGs were identified (table S1). These findings suggest that conventional threshold values may overlook highly divergent yet potentially functional phage-encoded ARGs. On the basis of these findings, using lenient threshold search strategies and PCM methods in 26,697 phage genomes available in NCBI, we identified 11,665 potential ARGs located on 4989 phage genomes (18.69% of the total), including 9382 ARGs identified by BLASTp showing homology to known ARGs, 9130 potential ARGs identified through HMMScan screening of conserved ARG domains, and 1870 potential ARGs identified through PCM based on their structural similarities (Fig. 1A and data S1). Of these potential ARGs, 98.63% (11,505 of 11,665) exhibit less than 70% amino acid sequence identity with previously reported ARGs. A total of 1632 potential ARGs were coidentified by these three selected approaches, where 91.12% (1487 of 1632) were classified as potential trimethoprim-resistance dihydrofolate reductase *dfr* genes (Fig. 1B and fig. S1).

These potential ARGs can be divided into six functional groups, including 1687 proteins that inactivate antibiotics, 227 proteins that participate in antibiotic efflux, 7265 proteins that affect the antibiotic target, 327 proteins that protect the antibiotic target, and 2106 proteins that replace the antibiotic target (Fig. 1A and data S1). These potential ARGs belong to more than 40 classes, conferring potential resistance to at least 13 antibiotic families: β-lactams (SBLs and MBLs), aminoglycosides [APH, AAC, AAC(6)-APH(2), SAT, and ANT], chloramphenicol (Cat), fosfomycin (FosB2), lincosamide (Lnu), macrolides (ErmB and MPH), para-aminosalicylic acid (ThyA), quinolone (Qnr), rifamycin (RpoB2), tetracyclines [TetM/O/W and Tet(50)], trimethoprim (Dfr), streptogramin (Van), and peptide antibiotics (pmr phosphoethanolamine transferase, Cls, MCR, and RpoC) (data S1). Among all these potential ARGs, genes encoding lactamase (SBLs and MBLs), thyA, Fluor_Res_DNA_Topo, and Dfr account for 87.44% (Fig. 1A). Only 138 ARGs carried by 75 phages had high identity (identity ≥90%) and high query coverage (query coverage ≥85%) with previously reported bacterial ARGs (Fig. 1C). These results indicate that only 0.28% (75 of 26,697) of the phage genomes analyzed carried reported ARGs, supporting that phages rarely carry reported bacterial-borne ARGs. Our data showed a 1.5-fold higher proportion of phages carrying reported ARGs than previously reported (0.18%, 3 of 1642) (*14*).

### Functional *dfrA* genes are enriched in lytic phages

**Fig. 2.**
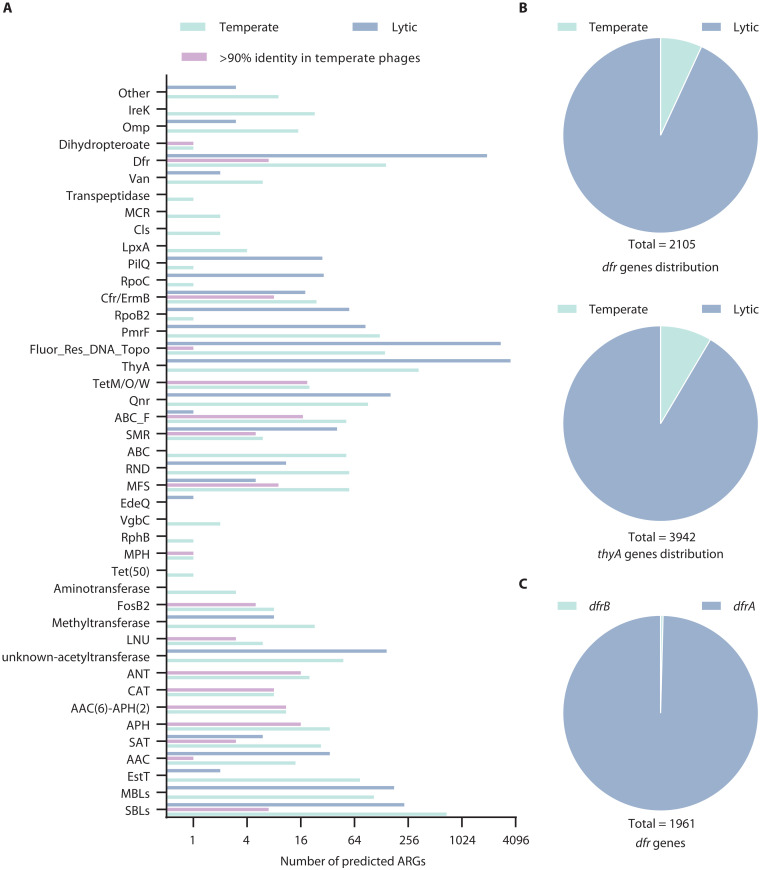
Overview of potential ARG distributions in lytic and temperate phages and analysis of potential *dfr* genes enrichment in lytic phages. (**A**) Distribution of the different types of potential ARGs profiles in temperate and lytic phages as predicted by BACPHLIP and PhaTYP analysis. Overview of potential ARGs with >90% identity to reported ARGs in temperate phages. (**B**) Distribution of potential *dfr* and *thyA* genes in lytic and temperate phages. (**C**) Distribution of potential *dfrA* and *dfrB* genes in lytic phages.

Multiple functional ARGs have been found in prophages (*10*, *11*) and temperate phage genomes (*12*). However, no functional ARGs have been identified in lytic phages (*12*). To analyze whether the candidate ARGs identified here are encoded in lytic or temperate phages, we used PhaTRY and BACPHLIP to predict the lifestyles of all phages as described in Materials and Methods and found that a total of 2246 potential ARGs were encoded by 1596 temperate phages, accounting for 18.80% (1596 of 8490) of all temperate phages, while 9419 potential ARGs were encoded by 3394 lytic phages, accounting for 18.64% (3394 of 18,207) of all lytic phages (data S1). Then, we analyzed the 138 reported ARGs in bacteria encoded by 75 phage isolates and revealed that all these reported ARGs were located on temperate phage genomes (Fig. 2A). These data further support that lytic phages rarely carry reported bacterial-borne ARGs. The analysis of the distribution of different types of predicted ARGs in lytic phages showed that *dfr* and *thyA* (thymidylate synthase) were notably enriched, showing 13- and 10-fold higher proportions than those found in temperate phages, respectively (Fig. 2, B and C).

Expression of *dfr* genes in bacteria confers resistance to trimethoprim (*17*). Trimethoprim binds to the dihydrofolate reductase (DHFR) encoded by the bacterial *folA* gene, inhibiting the conversion of dihydrofolic acid into tetrahydrofolic acid involved in the synthesis of thymine and purines (*17*). The *dfr* gene encodes an alternative DHFR protein that is insensitive to trimethoprim, leading to resistance. On the basis of their sequence and structural characteristics, the *dfr* genes were classified into *dfrA* and *dfrB* families. The *dfrA* family encodes a variant of the FolA protein conformed by 152 to 263 amino acids (*18*), while *dfrB* encodes a DHFR protein composed of ~78 amino acids, lacking sequence and structural similarity to FolA or DfrA (*19*).

**Fig. 3.**
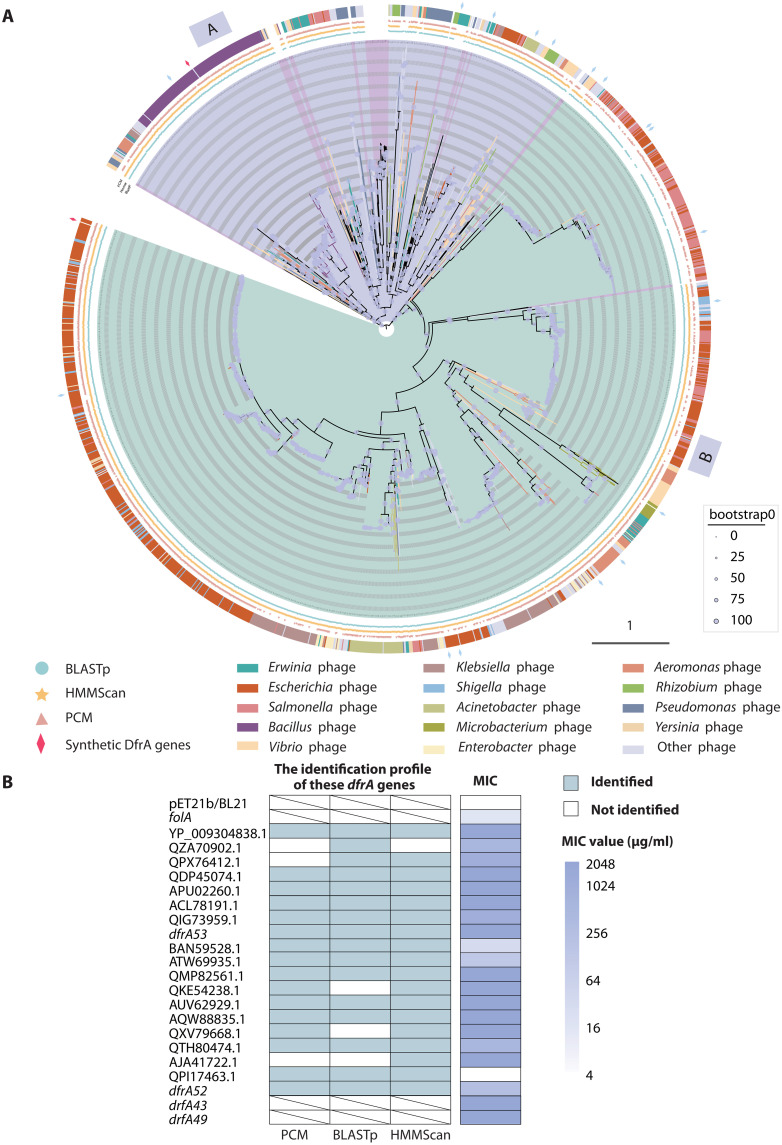
Phylogenetic analysis of potential DfrAs encoded by lytic phages and other reported DfrAs, and trimethoprim susceptibility profile of *E. coli* BL21(DE3) cells expressing random selected potential *dfrA* genes that were cloned from lytic phages. (**A**) Maximum likelihood phylogenetic tree of potential DfrAs encoded by lytic phages or reported DfrAs; this tree is rooted at midpoint and reveals two major clades A and B, distinguished by different background colors. The outer ring categorizes lytic phages based on their host classification. The three inner rings depict their identification as candidate DfrA through: PCM (pale pink triangle), HMMScan (orange-yellow pentagon), and BLASTp (light blue circle). Previously reported DfrAs are highlighted in red font. Diamonds on the outer ring indicate the random selected DfrA homologs encoded by lytic phages that were selected for expression and functional analysis. Red diamonds are DfrA52 encoded by *E. coli* phage vB_EcoM_BMB16 and DfrA53 encoded by *Bacillus* phage vB_BtM_BMBsp2, which were used for subsequent functional studies. (**B**) The minimum inhibitory concentration (MICs) of trimethoprim in *E. coli* BL21(De3) expressing *dfrA* genes cloned in pET21b(+) from randomly selected lytic phages. Empty vector, *folA*, and *dfrA49* were used as negative and positive controls, respectively. Figure for these results is shown as a heatmap.

We conducted a detailed analysis of 1961 potential *dfr* genes carried by 1952 lytic phages. Only eight candidate *dfr* genes belong to the *dfrB* family (Fig. 2D), showing a coverage range of 60 to 80% and an identity range of 50 to 72% compared to known DfrBs. The remaining 1953 candidate *dfr* genes, carried by 1944 lytic phages, showed less than 50% identity with reported *dfrA* genes (data S1). Construction of a phylogenetic tree of all potential DfrAs revealed that these proteins are grouped in two main branches, clade A and clade B, where most of the reported DfrAs cluster in clade A, with about one-third of the potential DfrAs encoded by lytic phages from *Bacillus*, *Microbacterium*, *Erwinia*, *Pseudoalteromonas*, etc. In contrast, most of the potential DfrAs (two-thirds of the remaining potential DfrAs) were found in clade B, encoded by lytic phages with different hosts such as *Escherichia*, *Salmonella*, *Enterobacteria*, *Klebsiella*, *Shigella*, etc. (Fig. 3A). These data show that lytic phages contain abundant potential *dfrA* genes.

To validate the functionality of potential *dfrA* genes identified through lenient threshold search strategies, we randomly selected 19 candidates from different subbranches of the phylogenetic tree to perform functional analysis. These genes were synthesized and expressed in *Escherichia coli* (see Materials and Methods). A total of 89.47% (17 of 19) of the potential *dfrA* genes conferred resistance to trimethoprim when expressed in *E. coli* (Fig. 3B and table S2). These results further demonstrate that lenient threshold strategies are effective for uncovering additional functional ARGs encoded by phage genomes, many of which would likely be missed under stringent criteria.

### Limited spread of lytic phage–borne *dfrA* genes

To assess the transmission risk of potential *dfrA* genes encoded by lytic phages, we analyzed the host of 1944 lytic phages carrying potential *dfrA* genes. These phages could infect bacteria belonging to 27 families of 44 genera, primarily Enterobacteriaceae, Bacillaceae, and Erwiniaceae (fig. S2). Also, nearly 91% of potential *dfrA* gene–carrying phages could infect Gram-negative bacteria, including major pathogens such as *Escherichia*, *Salmonella*, *Pseudomonas*, and *Klebsiella*. About 9% of the potential *dfrA* gene–carrying phages could infect Gram-positive bacteria like *Micrococcus* and *Bacillus* (fig. S2).

**Fig. 4.**
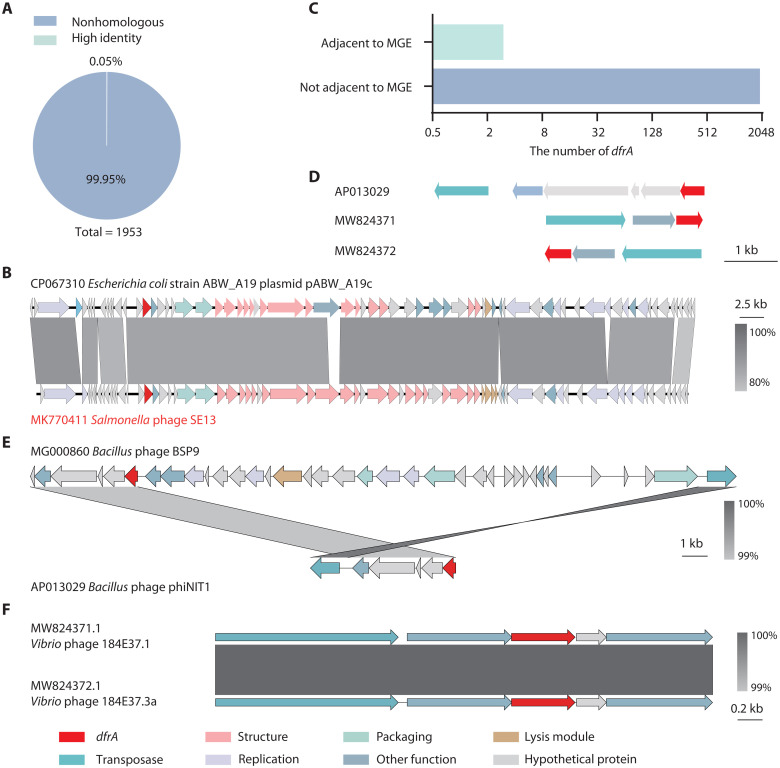
Lytic phages rarely spread their encoded potential *dfrA* genes. (**A**) Analysis of the number of potential *dfrA* genes from lytic phages that exhibit high sequence identity with bacterial genes. Only one gene from a total of 1953 potential *dfrA* genes encoded by 1944 lytic phages shows high sequence identity with bacterial genes (BLASTn >90% identity more than >90% of the query length). (**B**) Whole-genome alignment analysis of the potential lytic SE13 (MK770411) *Salmonella* phage carrying a *dfrA* gene (protein accession, YP_009845524.1) revealed high identity with a gene (protein accession, QTI37399.1) found in *E. coli* ABW_A19 strain pABW_A19c (CP067310) plasmid. The direction and size of the arrows indicate direction and size of the corresponding genes. (**C**) Analysis of the number of potential *dfrA* genes carried by lytic phages that were found adjacent or not adjacent to mobile elements. (**D**) Genomic organization of the three lytic phages with mobile elements adjacent to the potential *dfrA* genes. (**E**) Localized whole-genome comparison of *Bacillus* BSP9 and *Bacillus* phiNIT1 phages revealed that the transposase gene adjacent to potential *dfrA* gene in phiNIT1 phage has undergone HGT, whereas the potential *dfrA* gene itself has not been disseminated. The direction and size of the arrows indicate direction and size of the genes. (**F**) Localized whole-genome comparison of *Vibrio* phage 184E37.1 (MW824371.1) and *Vibrio* phage 184E37.3a (MW824372.1) showed that the two additional candidate *dfrA* genes were consistently linked to transposase genes. The direction and size of the arrows indicate direction and size of the genes.

The bacterial GenBank Nucleotide database (16 November 2024) was searched for homologous sequences to the 1953 potential *dfrA* genes carried by 1944 lytic phages using stringent criteria (BLASTn > 90% identity more than >90% of the query length). Only one potential *dfrA* gene was identified (Fig. 4A), corresponding to a lytic phage genome that exhibited high identity with a bacterial plasmid (Fig. 4B). These results imply that lytic phages rarely spread their encoded potential *dfrA* genes into their bacterial hosts.

Many ARGs found in plasmids are frequently flanked by mobile genetic elements (MGEs), such as insertion sequences (ISs) and transposons, which facilitate HGT transfer of ARGs among plasmids and chromosomes (*20*–*22*). A small number of phages have been reported to carry MGEs (*23*). Furthermore, ARGs in phage-plasmids are frequently adjacent to MGEs, potentially facilitating HGT of ARGs (*11*). We analyzed the presence of MGEs (transposases, conjugative elements, and integrons) in lytic phages carrying potential *dfrA* genes. We found that only three potential *dfrA* genes colocalized with a transposase homolog (Fig. 4, C and D). Further analysis revealed that in one potential *dfrA*-transposase module, only the transposase underwent independent HGT and was independent of its adjacent potential *dfrA* gene (Fig. 4E and fig. S3). In contrast, the other two potential *dfrA* genes were consistently linked to transposase genes and were exclusively found within phage genomes (Fig. 4F). These findings suggest that the likelihood of lytic phage–mediated potential *dfrA* gene propagation through MGEs is limited.

### Lytic phage–borne *dfrA* genes facilitate phage reproduction in the presence of trimethoprim

**Fig. 5.**
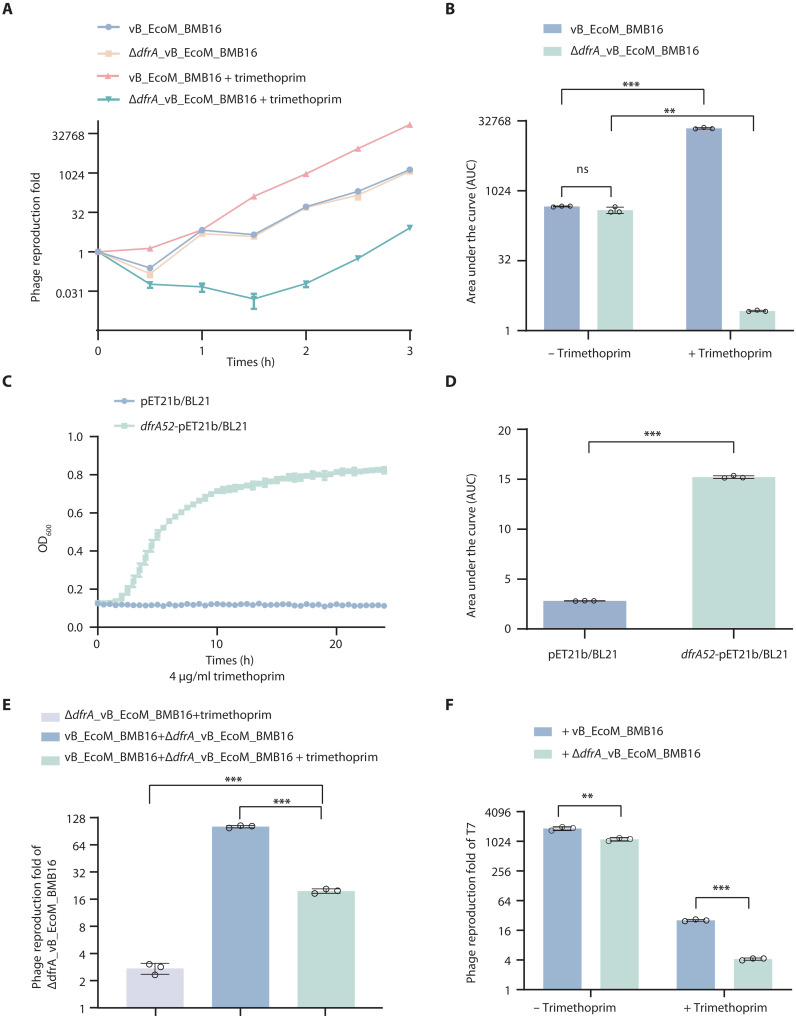
Lytic phage–borne *dfrA* genes facilitate phage reproduction in the presence of trimethoprim. (**A**) Reproduction of vB_EcoM_BMB16 and Δ*dfrA*_vB_EcoM_BMB16 phages in *E. coli* DH5α cells over 3 hours (h), with phage titers measured every 30 min under the presence or absence of trimethoprim (4 μg/ml). Phage reproduction fold, the phage titer at each time point divided by phage titer at 0 hours. Data represent the means ± SD of *n* = 3 biological replicates. (**B**) Quantification of the area under the curve (AUC) for (A), followed by statistical analysis. Data represent the means ± SD of *n* = 3 biological replicates. (**C**) Growth curves of *E. coli* BL21 strain expressing the *dfrA52* gene from vB_EcoM_BMB16 phage or transformed with empty vector as negative control under trimethoprim (4 μg/ml). Both strains were supplemented with ampicillin (100 μg/ml) and 0.2 mM β-d-1-thiogalactopyranoside (IPTG). Representative images of three independent replicates are shown. (**D**) Quantification of AUC for (C), followed by Welch’s *t* test for statistical analysis (*P* = 0.000027), as Levene’s test indicated unequal variance (*P* < 0.05). (**E**) Reproduction of Δ*dfrA*_vB_EcoM_BMB16 phage after 2 hours of culture under trimethoprim (4 μg/ml) or cocultured with vB_EcoM_BMB16 phage in the presence or absence of trimethoprim (4 μg/ml). Data represent the means ± SD of *n* = 3 biological replicates. (**F**) Reproduction of phage T7 after cultured with vB_EcoM_BMB16 or Δ*dfrA*_vB_EcoM_BMB16 phages for 2 hours under normal or trimethoprim (4 μg/ml) stress conditions. Data represent the means ± SD of *n* = 3 biological replicates. Statistical significance was determined using appropriate tests (see Material and Methods). Statistical significance thresholds: *P* > 0.05 (ns), **P < 0.01, ***P < 0.001.

Since DfrA synthesizes tetrahydrofolate, essential for DNA replication, and confers resistance to trimethoprim, we analyzed its role in phage replication in the presence of trimethoprim. The function of *dfrA* from lytic phage vB_EcoM_BMB16 expressing a functional *dfrA52* gene (encoding XPO54507.1) was evaluated in *E. coli* (Fig. 3B and table S1). Reverse transcription polymerase chain reaction (RT-PCR) and quantitative RT-PCR (qRT-PCR) expression analysis showed that the *dfrA52* gene was transcribed in infected *E. coli* cells (fig. S4, A and B). A *dfrA52* knock-out mutant was constructed by CRISPR-Cas9 (Δ*dfrA*_vB_EcoM_BMB16) as described in Materials and Methods (fig. S5, A to C). In the absence of trimethoprim, both wild-type vB_EcoM_BMB16 and Δ*dfrA*_vB_EcoM_BMB16 phages showed similar phage titers after *E. coli* infection (Fig. 5, A and B). In contrast, in the presence of trimethoprim, the reproduction of Δ*dfrA*_vB_EcoM_BMB16 was substantially inhibited, whereas vB_EcoM_BMB16 exhibited a notable increase in reproduction after *E. coli* infection (Fig. 5, A and B). These data indicate that the *dfrA52* gene does not affect phage reproduction in the absence of trimethoprim conditions but aids phage reproduction under trimethoprim pressure. These results suggest that the *dfrA52* gene carried by lytic phages helps host bacteria to survive under trimethoprim pressure, benefiting phage reproduction and possibly other phages infecting host bacteria.

To test this hypothesis, we analyzed the reproductive ability of phages when vB_EcoM_BMB16 harboring *dfrA52* was coinoculated with other lytic phages lacking *dfrA* in the presence of trimethoprim. First, we confirmed that the expression of *dfrA52* conferred trimethoprim resistance to *E. coli* (Fig. 5, C and D) and further demonstrated that this resistance was not mediated by the degradation of trimethoprim (fig. S6). Subsequently, we mixed vB_EcoM_BMB16 and Δ*dfrA*_vB_EcoM_BMB16 mutant and measured the reproduction of Δ*dfrA*_vB_EcoM_BMB16 in the presence of trimethoprim. Compared to infections with only Δ*dfrA*_vB_EcoM_BMB16, its reproductive ability in the presence of trimethoprim increased approximately sevenfold when it was mixed with vB_EcoM_BMB16 (Fig. 5E). Similarly, we mixed vB_EcoM_BMB16 and Δ*dfrA*_vB_EcoM_BMB16 with phage T7, whose reproduction was substantially inhibited by trimethoprim (fig. S7). Figure 5F shows that the reproduction of phage T7 increased ~sixfold in the presence of trimethoprim when vB_EcoM_BMB16 is present. These results indicate that the *dfrA52* gene carried by vB_EcoM_BMB16 helps the reproduction of Δ*dfrA*_vB_EcoM_BMB16 and phage T7 in the presence of trimethoprim, supporting that the *dfrA* genes carried by lytic phages help phage reproduction by conferring trimethoprim resistance to host bacteria.

### The *dfrA* gene carried by lytic phage also enhances host bacteria growth under phage infection

**Fig. 6.**
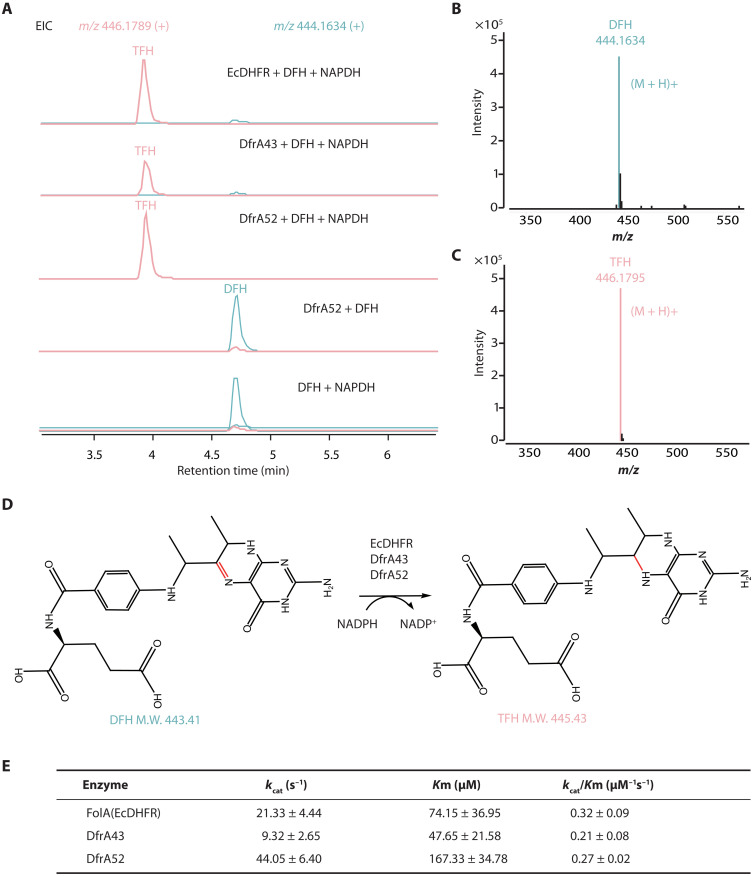
Lytic phage–borne DfrA has DHFR activity, which can convert dihydrofolate into tetrahydrofolate. (**A**) HPLC-MS analysis in vitro assay of the reaction products generated by EcDHFR, DfrA43, and DfrA52 acting on dihydrofolate (DHF) in the presence of reduced form of nicotinamide adenine dinucleotide phosphate (NADPH) as a coenzyme. The extracted ion chromatogram (EIC) traces of DFH [mass/charge ratio (*m*/*z*) 444.1634 (**B**)] and tetrahydrofolate (TFH) (*m*/*z* 446.1789) and under different reaction conditions. Positive MS spectra of TFH (B) and DFH (**C**), respectively. (**D**) Biosynthetic pathway of DHFRs (EcDHFR, DfrA43, and DfrA52) catalyzing the reduction of DHF to THF. (**E**) Kinetic parameters for the hydrolysis of dihydrofolate of DHFRs (EcDHFR, DfrA43, and DfrA52) under NADPH condition. The enzyme kinetics were analyzed by measuring the rate of decrease in absorbance at 340 nm (corresponding to NADPH oxidation) at different concentrations of dihydrofolate. Nonlinear regression analysis was performed to derive key parameters, including the Michaelis-Menten constant (*K*_m_) and the maximum reaction velocity (*V*_max_). The SE (±) was determined from three independent replicates.

Phage-encoded auxiliary metabolic genes can participate in both host and phage physiological activities. For instance, the *dap1* gene carried by the *Pseudomonas aeruginosa* PaoP5 phage not only contributes to phage fitness but also modulates host bacteria behavior (*24*). Given that DfrA52 exhibited DHFR activity, converting dihydrofolate into tetrahydrofolate, which is an essential component in various biosynthetic pathways such as amino acid and nucleic acid metabolism (Fig. 6) (*25*), we speculated that the *dfrA* gene, as an auxiliary metabolic gene, could also directly benefit host bacteria.

**Fig. 7.**
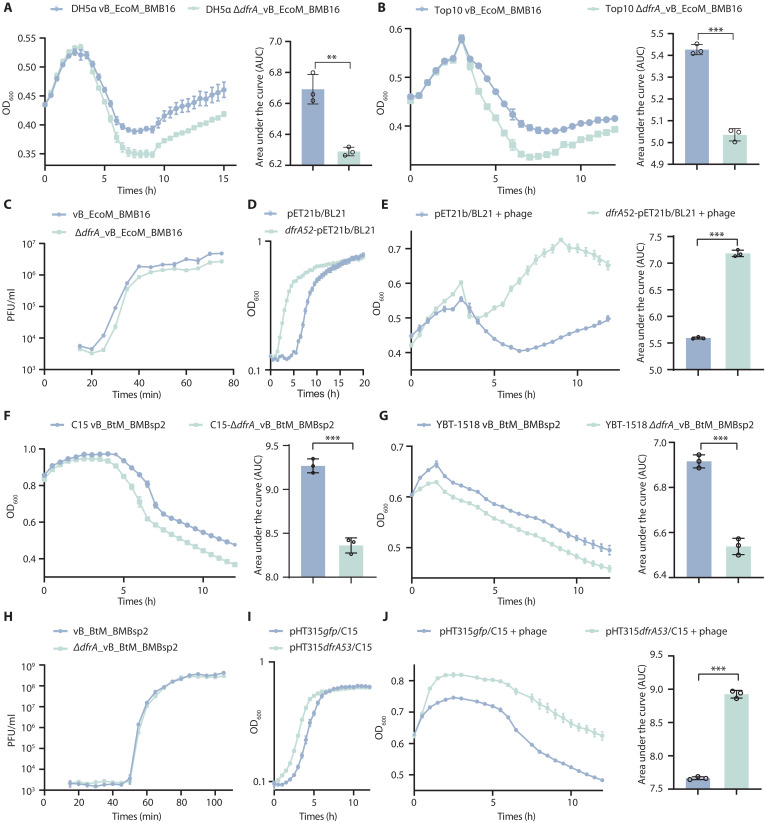
Lytic phage–borne *dfrA* genes enhanced the growth of host bacteria under phage infection. (**A** and **B**) Growth curves of *E. coli* DH5α (A) and Top10 strains (B) after infection with vB_EcoM_BMB16 and Δ*dfrA*_vB_EcoM_BMB16 phages at MOI = 0.1. AUC was quantified. (**C**) One-step growth curves of vB_EcoM_BMB16 and Δ*dfrA*_vB_EcoM_BMB16 phages infecting *E. coli* DH5α. The latent period was 20 min (15 min adsorption + 5 min), with burst sizes of 394.81 ± 10.50 and 370.70 ± 15.25 PFUs per cell, respectively (*n* = 3, *P* = 0.087, *t* test). (**D**) Growth curves of *E. coli* BL21(DE3) strain expressing *dfrA52* gene from vB_EcoM_BMB16 phage or containing empty vector as negative control after 0.2 mM IPTG induction. (**E**) Growth curves of *E. coli* BL21(DE3) strain expressing *dfrA52* gene from vB_EcoM_BMB16 phage or containing empty vector as negative control; both strains were infected with Δ*dfrA*_vB_EcoM_BMB16 phage at MOI of 0.1 under 0.2 mM IPTG induction. AUC was quantified. (**F** and **G**) Growth curves of *B. thuringiensis* C15 (F) and YBT-1518 (G) strains after infection with vB_BtM_BMBsp2 or Δ*dfrA*_vB_BtM_BMBsp2 phages at MOI of 0.1. AUC was quantified. (**H**) One-step growth curves of vB_BtM_BMBsp2 and Δ*dfrA*_vB_BtM_BMBsp2 phages infecting *B. thuringiensis* C15 strain. The latent period was 45 min (15 min adsorption + 30 min). The burst sizes were 151,893.49 ± 20,690.94 and 127,465.66 ± 16,413.71 PFUs per cell, respectively (*n* = 3, *P* = 0.184, *t* test). (**I**) Growth curves of C15 strain expressing *dfrA53* gene from vB_BtM_BMBsp2 and containing empty vector. (**J**) Growth curves of *B. thuringiensis* C15 strain expressing *dfrA53* or *gfp* genes; both strains were infected with Δ*dfrA*_vB_BtM_BMBsp2 phage at an MOI of 0.1. AUC was quantified. Data represent the means ± SD of *n* = 3 biological replicates. Statistical significance was determined using appropriate tests (see Material and Methods).

We first compared the growth of *E. coli* host after infection with vB_EcoM_BMB16 or Δ*dfrA*_vB_EcoM_BMB16 phages under normal conditions and found that Δ*dfrA*_vB_EcoM_BMB16 exhibited stronger antibacterial activity (Fig. 7, A and B, and fig. S8, A and B). The *dfrA52* gene did not affect phage reproduction, plaque size, or phage morphology (Fig. 5, A and B, and fig. S9, A to F). Also, the one-step growth curve experiment showed that the *dfrA52* gene did not affect the latent period and burst sizes (Fig. 7C). We speculated that the production of tetrahydrofolate by DfrA52 enhanced the synthesis of DNA precursors, promoting cell proliferation. The heterologous expression of *dfrA52* in *E. coli* shortened the lag phase of bacterial growth, and increased colony-forming units (CFUs) during the logarithmic growth phase (Fig. 7D and fig. S8C). In addition, host bacteria overexpressing the *dfrA52* gene showed increased survival after infection with Δ*dfrA*_vB_EcoM_BMB16 phage (Fig. 7E and fig. S8D). Furthermore, under conditions of low bacterial density, the Δ*dfrA*_vB_EcoM_BMB16 phage markedly suppressed bacterial growth, in contrast to bacterial populations infected with the vB_EcoM_BMB16 phage, which showed a rapid resumption of growth 12 hours postinfection (fig. S10, A to D). These findings showed that the *dfrA52* gene helps the host bacteria to grow after phage infection, potentially alleviating the growth pressure imposed by the phage.

Furthermore, the effect of the *dfrA53* (encoding UJH95751.1) gene in the *Bacillus* lytic vB_BtM_BMBsp2 phage was analyzed. First, RT-PCR and qRT-PCR confirmed that the *dfrA53* gene was transcribed after vB_BtM_BMBsp2 phage infection (fig. S4, C and D). Subsequently, we used the CRISPR-Cas9 system to create a *dfrA53* deletion mutant, Δ*dfrA*_vB_BtM_BMBsp2 (fig. S5, D to F). Since host *Bacillus thuringiensis* exhibits high tolerance to trimethoprim (MIC >512 μg/ml), the *dfrA53* gene did not affect phage reproduction under trimethoprim conditions. Similar to *dfrA52*, the deletion of *dfrA53* also exhibited stronger antibacterial activity compared with wild phage (Fig. 7, F and G, and fig. S8, E and F). The deletion of *dfrA53* does not affect plaque size, phage morphology, phage latent, and burst size (fig. S9, J to L, and Fig. 7H). In addition, the expression of the *dfrA53* gene also shortened the lag phase of bacterial growth and increased CFUs during the logarithmic growth phase (Fig. 7I and fig. S8G). Also, the survival rate of host bacteria expressing the *dfrA53* gene was substantially improved after infection with the Δ*dfrA*_vB_BtM_BMBsp2 phage (Fig. 7J and fig. S8H). Similarly, under low bacterial density conditions, the extent of bacterial population recovery following vB_EcoM_BMB16 phage infection was greater than that observed under Δ*dfrA*_vB_EcoM_BMB16 mutant-phage infection (fig. S10, E to H).

## DISCUSSION

Phages, the most abundant biological entities on earth, are considered important MGEs for HGT (*26*–*28*). Known ARGs had already been identified in prophages, temperate phages, and multiple viral fractions (*15*, *29*–*32*) but not in lytic phages (*12*–*14*). Considering the identification of a small number of functional ARGs in the virome not related to bacterial-borne ARGs (*15*), we adopted lenient threshold search strategies and PMC methods based on machine learning of structural similarity to systematically analyze the presence of potential ARGs in all phage isolates. Although we cannot discard some false positive results, our findings indicate that lytic phages are a reservoir of functional *dfrA* genes that exhibit notable sequence differences with bacteria-borne *dfrA* genes. However, we show here that lytic phages did not spread these potential *dfrA* genes to bacteria. We found that expression of *dfrA* gene during infection, in the presence of trimethoprim, allows lytic phage reproduction. In addition, *dfrA* expression improved host bacteria growth, suggesting a positive selection for *dfrA* genes during the interaction of lytic phages with their bacterial hosts.

The trimethoprim/sulfamethoxazole antibiotics are recommended by the World Health Organization for the treatment of urinary and respiratory tract infections caused by a variety of pathogens, such as *Klebsiella pneumoniae*, *Salmonella enterica*, and *E. coli* (*33*, *34*). Only one homolog potential *dfrA* gene encoding QDH45123.1 carried by *Salmonella* lytic phage SE13 was identified in bacteria (Fig. 4B), suggesting its dissemination into bacterial populations. However, this gene (protein accession: QTI37399.1) was exclusively found in an *E. coli* strain ABW_A19 plasmid pABW_A19c (CP067310) (Fig. 4B), which exhibited a high degree of identity (coverage 99% and homology >95.78%) with the typical lytic phage *Salmonella* phage vB_SenM-1 (*35*), and lacked typical genes associated with the lysis/lysogeny switch and plasmid replication–related genes. Unexpectedly, plasmid pABW_A19c exclusively harbored phage-related elements without plasmid replication–related elements. Therefore, this result suggests that the *E. coli* ABW_A19 plasmid pABW_A19c was derived from a pseudo-lysogeny state of *Salmonella* phage vB_SenM-1. Previous studies have shown that the pseudo-lysogeny state of lytic phages exhibits low stability, ultimately transitioning to virulent growth (*36*). Therefore, we inferred that the likelihood of *Salmonella* phage SE13 disseminating its potential *dfrA* gene extensively in nature through a pseudo-lysogenic state is limited.

The replication process of phages depends on host cells (*37*). Unlike temperate phages, lytic phages cannot integrate into the host bacterial genome to replicate within the host genome. Instead, after infecting the host bacteria, lytic phages use the genetic machinery of the host to proliferate. We show here that the *dfrA* gene encoded by lytic phages enhanced the growth of host bacteria in the early stages of phage infection (Fig. 7). This phenotype differs from other auxiliary metabolic genes carried by phages that benefit phage proliferation by promoting the growth and metabolism of host bacteria (*38*, *39*). We speculate that in the early stages of lytic phage infection, when the number of host bacteria in the environment is limited, the expression of *dfrA* helps the host bacteria to multiply rapidly, preventing the phage from eliminating all host bacteria in the environment. This phenomenon reflects a mutualistic evolutionary relationship between lytic phages and their hosts and represents a previously unreported survival strategy for lytic phages.

Our analysis showed that there are no mobile elements adjacent to potential *dfrA* genes except for three potential *dfrA* genes (Fig. 4, C to F). Given that the potential *dfrA* genes in lytic phages were not acquired through HGT, that the amino acid sequences of the proteins they encode were less than 50% identical to reported bacterial DfrAs (data S1), and that previous studies have shown that the *dfrA* genes in bacteria originated from archaeal ancestors (*40*), we speculate that the *dfrA* genes in bacteria and lytic phages evolved independently. In addition, these potential *dfrA* genes carried by lytic phages provided an advantage to the phage and the host bacteria (Fig. 5 and Fig. 7). This suggests that the main driving force of selection of potential *dfrA* genes in lytic phages was to improve host fitness.

In addition to the potential *dfrA* genes, we have identified several other potential ARGs within lytic phages. While further studies are necessary to confirm whether these potential ARGs can confer antibiotic resistance to bacteria, we evaluated the risk of lytic phages transmitting these ARGs. Our analysis showed that 29.79% (669 of 2246) of potential ARGs carried by temperate phages spread to bacterial populations, whereas only 0.46% (43 of 9419) of potential ARGs carried by lytic phages were found disseminated among bacteria (fig. S11 and data S2). These findings indicate that temperate phages are highly likely to transmit potential ARGs, whereas potential ARGs carried by lytic phages have a low transmission rate. Subsequently, we analyzed whether mobile elements were adjacent to these potential ARGs. Only 0.14% (13 of 9419) of potential ARGs carried by nine lytic phages have adjacent MGEs, including the potential *dfrA* gene (fig. S12). In addition, among the potential ARGs carried by temperate phages, 5.30% (119 of 2246) have adjacent MGEs carried by 60 temperate phages (fig. S13). These findings indicated that the potential dissemination of potential ARGs carried by lytic phages through MGEs is low.

Our data show that there is a substantial difference in the transmission probability of potential ARGs carried by lytic phages and temperate phages. This could be due to the different lifestyles of these two phage classes. Lytic phages rapidly replicate and lyse the host bacteria, which reduces the chance of genetic material exchange between the lytic phages and their host bacteria. In contrast, temperate phages have the capacity for lysogeny, facilitating their genome integration into host bacteria genome (*41*, *42*). During this process, potential ARGs are concomitantly transferred to the host bacterial genome, thereby conferring antibiotic resistance (*43*). The transmission risk of potential ARGs carried by lytic phages appears relatively low. However, lytic phages can spread bacterial ARGs through generalized transduction (*7*, *44*). The transducing particles lack the phage’s own genetic material, so lytic phages will not spread their own ARGs through generalized transduction (*44*, *45*). Nevertheless, we found a few cases (0.46%) where potential ARGs encoded by lytic phages were transmitted to bacteria (fig. S10), which requires further investigation.

We found that expression of the *dfrA52* gene, derived from a lytic phage, conferred trimethoprim resistance to the host bacterium (Fig. 5C). Quantitative analysis by high-performance liquid chromatography–mass spectrometry (HPLC-MS) further confirmed that the concentration of trimethoprim in the culture medium remained unchanged under *dfrA52* expression (fig. S6), indicating that the resistance mechanism does not involve drug degradation. These findings are consistent with the well-characterized mechanism of *dfrA*-mediated trimethoprim resistance (*17*), whereby a resistant DHFR enzyme, insensitive to trimethoprim inhibition, replaces the native enzyme, thereby maintaining folate metabolism and conferring resistance.

We acknowledge that the identity threshold applied in our analysis is, to some extent, subjective. While more lenient thresholds may increase the risk of false positives, they enabled the identification of a broader range of potential ARGs, particularly those that are highly divergent from known sequences. Our sensitivity analysis showed that applying stricter thresholds substantially reduced the number of detectable ARG candidates, especially in lytic phages (table S1). Moreover, functional validation demonstrated that 17 of 19 randomly selected *dfrA* candidates conferred trimethoprim resistance (table S2), providing strong support for the effectiveness of lenient threshold search strategies in uncovering additional functional ARGs that would likely be overlooked by conventional screening criteria.

In conclusion, our findings revealed that lytic phages can act as reservoirs of *dfrA* genes, which breaks the dogma that lytic phages rarely carry functional ARGs. Currently, phage therapy prioritizes lytic phages without ARGs to minimize ARG dissemination. However, our study demonstrates that lytic phages could seldom disseminate their encoded potential ARGs. This broadens the selection criteria for therapeutic phages, indicating that the presence of certain ARGs in lytic phages may not be a major concern. In addition, our findings demonstrate that lytic phage–borne *dfrA* genes not only help phage reproduction in the presence of trimethoprim but also enhance the growth of host bacteria under phage infection, reflecting the mutualistic evolutionary relationship between lytic phages and their hosts.

## MATERIALS AND METHODS

### ARGs search from phage genomes

The genome sequences of all phage isolates were downloaded from the PHAge REference Database (INPHARED) dataset (retrieved on 05 December 2024) (*12*), yielding a total of 31,787 phage genomes. During data processing, we observed that some phages shared the same name but had different accession numbers. To assess redundancy, we conducted a nucleotide identity analysis of these genomes. The results revealed that phage genomes with identical names but different accession numbers exhibited nucleotide identity and coverage greater than 99.9%. On the basis of this finding, we removed redundant genome entries, retaining a total of 26,884 unique phage genomes. In addition, we excluded mutant phages from the dataset, resulting in a final set of 26,697 phage genomes for analysis. The complete list of genomes and their corresponding accession numbers is provided in data S1. All protein sequences were extracted from phage genome sequences. Two different search strategies were used to identify the candidate ARGs from the proteins found in phage genomes. First, lenient threshold search strategies of potential ARGs were implemented according to the methods described by Moon *et al.* (*15*), with all phage protein sequences as queries using BLASTp (version 2.14.0) against the CARD database (*46*) with *E*-value thresholds ≤1 × 10^−5^ and query coverage ≥60%, and by using HMMScan (version 3.3.2) against the Resfams ARG database (*47*) with *E*-value threshold ≤1 × 10^−5^ and a score ≥40. Second, search strategies based on machine learning of structural similarity were executed using PCM framework as developed by Ruppe *et al.* (*16*) using default parameters. According to the definition of ARGs by Martínez *et al.* (*48*), ARGs refer to genes that encode proteins capable of conferring antibiotic resistance to bacteria, or the absence of which intensifies bacterial susceptibility to antibiotics. We manually excluded specific genes responsible for regulating the expression of ARG and those that induce bacterial resistance through mutation.

### The lifestyle prediction for all phage isolates

Phages can be classified into lytic and temperate phages based on their lifestyle. To better discern the lifestyles of all phages identified, we used two distinct approaches: the BACPHLIP (version 0.9.6) method, which is based on identifying the presence of a set of lysogeny-related protein domains (*49*), and the PhaTYP method using Bidirectional Encoder Representations from Transformer to learn protein composition and associations for classifying the lifestyles of phages (*50*). To accurately distinguish lytic phages, we defined all potential temperate phages predicted by BACPHLIP and PhaTYP as candidate temperate phages (data S1). Subsequently, we refined the predictions from PhaTYP and BACPHLIP through literature calibration and determined that seven potential lytic phages carrying potential ARGs initially identified by PhaTYP and BACPHLIP, named *Salmonella* phage SSU5 (JQ965645) (*51*), five high-identity *Vibrio cholerae* filamentous phage CTX (KJ540274, KJ540275, KJ540276, KJ540277, and KJ540278) (*52*), and filamentous phage cloning vector fd-tet (AF217317) (*53*), are indeed temperate phages based on the literature review. Previous studies have shown that the *parA* and *parB* genes, encoding plasmid-like partitioning proteins, can replace integration cassettes, allowing phage genome integration into the host and the maintenance of a lysogenic state (*54*, *55*). Thus, any phage containing *parA* and *parB*-like genes in its genome and integrated into a bacterial host genome can be designated as a temperate phage. To further determine the lifestyle of all phages, we used HMMsearch (version 3.3.2) to identify phage genomes encoding candidate proteins with ParA and ParB domains. A total of 1368 such phage genomes were detected across all analyzed phage genomes. Subsequently, BLASTn (version 2.14.0) was performed against the Bacterial GenBank Nucleotide database (retrieved on 16 November 2024) to determine whether the phage genomes exhibited a high degree of whole-genome alignment with bacterial genome (criteria set at >90% identity and >90% coverage). The results revealed that 16.23% (222 of 1368) of the phage genomes with ParA and ParB domains exhibited a high degree of whole-genome alignment with bacterial genomes. On the basis of these findings, we designated all phage genomes carrying *parA* and *parB* genes as potentially integrative and classified them as putative temperate phages.

### Phylogenetic tree construction of DfrA encoded by lytic phage

We collected all reported DfrA protein sequences from the literature and GenBank, including DfrA1-49 from Gram-negative bacteria (*18*, *56*), and DfrC-K from Gram-positive bacteria (available at https://ncbi.nlm.nih.gov/pathogens/refgene). Then, multiple sequence alignment of all potential lytic phage–borne DfrAs and previously reported bacterial DfrAs (see data S4) was performed using MAFFT v7.520 with specific parameters (--auto) (*57*). The alignments were trimmed using trimAl 1.4.1 with the automatic method based on similarity statistics (-gt 0.05) (*58*). The phylogenetic trees were constructed for all trimmed DfrAs using IQ-TREE 1.6.9 with 1000 ultrafast bootstrap replicates (-m TEST -alrt 1000 -bb 1000 parameters) (*59*). The host information of lytic phages carrying potential *dfrA* genes was retrieved from their respective GenBank annotations and cross-referenced with the NCBI Taxonomy database. For phages with explicitly annotated host information in GenBank, classification was directly assigned on the basis of these records. For phages lacking host annotations, the host genus was inferred from the phage name, as phage nomenclature typically reflects their host genus. Phages for which no host genus information could be determined were excluded from further analysis. The detailed host classification, including phage name, accession number, and host taxonomy (genus, family, order, class, phylum, kingdom, and superkingdom), was provided in data S3. The phylogenetic tree was visualized using iTOL (*60*), and its root was determined at the midpoint.

### Construction of plasmids and transformation of *E. coli* strains

We randomly selected 19 representative potential *dfrA* genes across different subclades of the phylogenetic analysis of the lytic phages. The codon usage of these gene sequences was optimized for expression in *E. coli* and commercially synthesized by AuGCT Biotech. We also synthesized *dfrA49* (RefSeq nucleotide accession NG_242636.1) and *dfrA43* (RefSeq nucleotide accession NG_070721.1) genes as positive controls. The open reading frame of *folA* gene (RefSeq nucleotide accession NP_414590.1), encoding chromosomal DHFR (protein accession AOO72573.1), was amplified using *E. coli* DH5α genome as template. The synthesized *dfrA* and *folA* genes were individually subcloned into the pET21b(+) vector previously digested with NdeI and XhoI, using homologous recombination (ClonExpress II One Step Cloning Kit, Vazyme). Clones were selected after verification by Sanger sequencing. The resulting recombinant vectors, along with the empty pET21b(+) vector, were extracted from *E. coli* DH5α and introduced into *E. coli* BL21(DE3) by chemical transformation method. Colonies were selected on ampicillin (100 μg/ml), and recombinant strains were identified by colony-PCR.

### Antimicrobial susceptibility testing

The minimum trimethoprim inhibitory concentrations (MICs) for *E. coli* recombinant strains expressing different candidate *dfrA* genes were obtained by broth macrodilution method in accordance with the Clinical and Laboratory Standards Institute guidelines (*61*). Specifically, we measured the growth of the recombinant strains in Mueller-Hinton broth supplemented with isopropyl β-d-1-thiogalactopyranoside (IPTG, 0.1 mmol/liter) and trimethoprim (ranging from 0.5 to 2048 mg/liter in doubling concentrations). Growth or absence of growth was determined by measuring the turbidity of the culture. The recombinant strains expressing *dfrA49*, *dfrA43*, empty vector, or expressing *folA* were used as positive and negative controls, respectively. The MIC experiments were conducted in triplicate.

### Analysis of the transmission of *dfrA* genes from lytic phages to bacterial hosts

We searched for the homologs of all lytic phage–borne *dfrA* genes from the GenBank nt database of all bacteria (up to 16 November 2024) based on the method by Ruppe *et al.* (*16*) using BLASTn (version 2.14.0) (>90% identity, more than >90% of the query length). For each identified homologous sequence, we further manually verified its origin, confirming its location either on the bacterial chromosome or plasmid rather than in the phage genome. This verification step was critical to minimize errors resulting from potential annotation inaccuracies in the bacterial nt database. The lytic phage–borne *dfrA* genes were considered to have undergone dissemination between lytic phages and bacteria when their homologs were detected in plasmids or in bacterial genomes. To further elucidate whether the disseminated lytic phage–borne *dfrA* genes were transferred independently or together with other phage genes into the bacterial hosts, the bacterial chromosomes or plasmids harboring the disseminated *dfrA* genes were identified by BLASTn (version 2.14.0). Then, whole-genome alignments between the lytic phage genomes and their bacterial hosts were constructed using EasyFig (version 2.2.3) (*62*).

### Analysis of the presence or absence of MGEs adjacent to the potential *dfrA* genes in lytic phages

We analyzed the mobile elements (including IS elements, conjugative elements, and integrons) in the genomes of all potential *dfrA* gene–containing lytic phages. All protein sequences encoded by lytic phages harboring potential *dfrA* genes were downloaded from the NCBI Nucleotide database using Batch Entrez. IS elements were identified using BLASTp (version 2.14.0) against the ISfinder database (*63*) (query size threshold, 150 amino acids; *E* value 1 × 10^−30^; identity, threshold >40%). Conjugative elements were retrieved by ConjScan software (*64*) (*E* value <0.001; sequence coverage ≥50%). Integrons from lytic phage genomes were detected by IntegronFinder software (version 2.0.2) (*65*) with default parameters. Identified MGEs-related protein candidates were further validated using NCBI’s Conserved Domain Database (CDD) (https://ncbi.nlm.nih.gov/Structure/cdd/wrpsb.cgi). Last, we manually analyzed the genomic locations of the mobile elements identified by the above software tools to determine whether they were adjacent to the potential *dfrA* genes (<5 genes intergenic intervals).

### Bacteria, bacteriophages, and their growth conditions

The bacterial strains, phages, and plasmids used in this study are detailed in data S1. All strains were cultured in LB medium (1% tryptone, 0.5% yeast extract, and 0.5% NaCl). *E. coli* was grown at 37°C and *B. thuringiensis* at 28°C, with shaking at 220 rpm or on LB agar plates (1.5% agar). When necessary, antibiotics were added to the culture medium [kanamycin (50 μg/ml), ampicillin (100 μg/ml), erythromycin (25 μg/ml), and spectinomycin 25 μg/ml)].

Phages were propagated by culturing host bacteria in LB liquid culture. *E. coli* DH5α cultures optical density at 600 nm (OD_600_) ~0.4 to 0.6 were infected with a multiplicity of infection (MOI) of 0.1 and incubated at 37°C for 6 to 8 hours with aeration. Similarly, *Bacillus* phages were propagated by infecting *B. thuringiensis* C15 cultures at an OD_600_ of ~0.4 to 0.6 at MOI of 0.1, 28°C for 6 to 8 hours with aeration. Subsequently, the remaining cells were then pelleted by centrifugation for 4 min at 12,000 rpm and the supernatant was filtered through a 0.22-μm pore size sterile Polyethersulfone (PES) filter to obtain the phage suspension.

### Phage plaque morphology, phage titer of plating assays

Overnight cultures of the indicated bacterial cells were transferred to fresh liquid culture medium at a 1% ratio and grown to an OD_600_ of ~0.4 to 0.6. The phage titer was determined using the double-layer overlay plate assay. Briefly, the phage suspension was diluted 10-fold in series, and then 100 μl of phages at different dilutions were mixed with 900 μl of bacterial culture and 5 ml of warm 0.5% agar LB medium. This mixture was then poured onto LB bottom layer plates containing 8 ml of 1.5% agar. Phage plaque morphology was observed using a Canon EOS RP. All experiments were performed independently at least three times.

### Phage isolate, sequencing, annotation, and morphology analysis

*E. coli* phage vB_EcoM_BMB16 and *Bacillus* phage vB_BtM_BMBsp2 were isolated from environmental soil samples using *E. coli* DH5α and *B. thuringiensis* YBT1518 as host bacteria, respectively. Phage isolation was performed as previously described (*66*). First, 2 g of soil sample was added to a 250-ml sterile conical flask containing 30 ml of LB. The culture was then incubated overnight at 28° or 37°C with continuous shaking at 200 rpm. Then, the incubated sample was centrifuged at 12,000 rpm and 4°C for 10 min, and the supernatant was filtered through a 0.22-μm pore size sterile PES filter to obtain a bacteria-free soil suspension. This soil suspension was incubated with *E. coli* DH5α or *B. thuringiensis* YBT1518 at OD_600_ of ~0.5 for 30 min. Last, phages infecting *E. coli* DH5α or *B. thuringiensis* YBT1518 were detected using spot-test assay with the double-layer agar method. Single phage plaques were picked from the double-layer agar plate using a sterile pipette and purified five times in succession to obtain pure single phage cultures.

The genomic DNA of phages vB_EcoM_BMB16 and vB_BtM_BMBsp2 was extracted using ZnCl_2_ precipitation. The extracted DNA was fragmented to an average size of 350 base pairs (bp) using the Covaris LE220R-plus system (Covaris, USA). Library preparation was performed following standard Illumina protocols, including end-polishing, A-tailing, adapter ligation, and PCR amplification. The amplified libraries were purified using the AMPure XP system (Beckman Coulter, USA), and library quality was analyzed using the Agilent 5400 system (AATI). The libraries were quantified by real-time PCR (final concentration 1.5 nM) and pooled for sequencing. Paired-end (PE150) sequencing was conducted using the Illumina HiSeq 2500 platform at Novogene Bioinformatics Technology Co. Ltd. (Beijing, China). Raw sequencing reads were quality-checked using FastQC (https://www.bioinformatics.babraham.ac.uk/projects/fastqc/), and low-quality bases (Q < 20) and adapter sequences were trimmed using Trimmomatic v0.39. Reads shorter than 50 bp after trimming were discarded. The genomes of vB_EcoM_BMB16 and vB_BtM_BMBsp2 were de novo assembled using ABySS v2.0 with multiple k-mer sizes (40, 45, 50, 55, 60, 65, and 70). Phage genome completeness was assessed on the basis of N50 values and the presence or absence of terminal repeat sequences. Last, the genomes were annotated using the online tool RAST Server version 2.0. The phage genomes have been deposited in NCBI: vB_EcoM_BMB16 (accession number PV102577) and vB_BtM_BMBsp2 (accession number OL964058.1).

The phage morphology of vB_EcoM_BMB16, vB_BtM_BMBsp2 and their *dfrA*-deletion mutants was visualized by negative staining under transmission electron microscopy as previously described (*67*). Briefly, these phage samples were placed on a carbon-coated copper grid. Then, they were negatively stained with 2% (w/v) phosphotungstic acid (pH 6.8) for 20 s. The stained grids were then air-dried and examined using an H-7650 transmission electron microscope at 100 kV in the Public Laboratory of Electron Microscopy, Huazhong Agricultural University (Wuhan, China). Morphological measurements, including capsid diameter and tail length, were calculated from micrographs of five randomly selected phage particles.

### Phage gene editing techniques using CRISPR-Cas9 system

To knockout *dfrA52* gene in *E. coli* phage vB_EcoM_BMB16 by CRISPR-Cas9 system, we used pCas9 and pTargetF plasmids (Addgene) in *E. coli*, as described before (*68*). Specifically, the single guide RNA (sgRNA) targeting *dfrA52* was designed by CRISPy-web (https://crispy.secondarymetabolites.org/#/input) (*69*) (see data S1 for details), primers Ec16-sgRNA-F/R were used to attach the sgRNA to the pTargetF vector via inverse PCR. The template was digested with *Dpn*I and transformed into *E. coli* DH5α, resulting in the vector *dfrA52*-sgRNA-pTargetF. Subsequently, primers Ec_16up-XbaI-F and Ec_16up-R and primers Ec_16down-F and Ec_16down-PstI-R were used to amplify the upstream 508 bp and downstream 500-bp fragments of the *dfrA52* gene, respectively. These fragments were then joined through Splicing by Overlap Extension (SOE) using the primers Ec_16up-XbaI-F and Ec_16down-PstI-R. The resulting product was digested with *Xba*I and *Pst*I and ligated into the *dfrA52*-sgRNA-pTargetF vector, digested with the same enzymes, creating the *dfrA52*-pTargetF vector containing both the sgRNA and repair arm. The vector, along with pCas9, was transformed into DH5α to generate *dfrA52*-pTargetF-pCas9/DH5α. After induction with 10 mM arabinose, the bacteria were infected with 10^5^ plaque-forming units (PFUs)/ml of phage vB_EcoM_BMB16 for 6 hours. Surviving single phage plaques were picked from the double-layer agar plate using a sterile pipette. PCR and Sanger sequencing were used to verify the presence of mutant phages. Specific primers, Ec_16_*dfr*-upup-F (5′-TAGCCATTACGCACATTA-3′) and Ec_16_*dfr*-dodo-R (5′-AAGATTCAGAAGTTAGAGGTA-3′) were used to amplify the target region with Easy Taq DNA polymerase. The PCR cycling conditions were as follows: initial denaturation at 95°C for 10 min, followed by 30 cycles of 95°C for 30 s (denaturation), 55°C for 30 s (annealing), and 72°C for 2 min (extension), with a final extension at 72°C for 10 min. The PCR products were subsequently purified and subjected to Sanger sequencing to verify the mutations (fig. S5, A and B). Sequencing results were aligned to the reference sequence to confirm the presence of the desired mutations. The mutant phage was purified continuously five times to obtain a pure phage mutant.

The *dfrA53* gene in the *Bacillus* phage vB_BtM_BMBsp2 was knocked out using the CRISPR-Cas9 system by using pJOE8999 plasmid as described in (*70*). The sgRNA targeting the *dfrA53* gene was designed by CRISPy-web (see data S1 for details) and was generated by annealing primers BMBsp2-sgRNA-F/R and ligated into the Eco31I-digested pJOE8999 plasmid. The upstream 655 bp and downstream 607-bp fragments of the *dfrA53* gene were then amplified using primers BMBsp2-up-SfiI-F/R and BMBsp2-down-F/R, respectively. These fragments were joined by SOE ligation, digested with SfiI, and subsequently ligated into the SfiI-digested pJOE8999 plasmid, creating the vector *dfrA53*-pJOE8999 containing both the sgRNA and repair arms. This vector was then transformed into YBT1518 to obtain *dfrA53*-pJOE8999/YBT1518. This strain was infected with 10^5^ phages, and pure phage mutants were obtained using the *E. coli* phage mutant screening method described above. To confirm the presence of phage mutations, PCR amplification and Sanger sequencing were performed using the primers BMBsp2_*dfrA*-upup-F (5′-ATGTCGTATGCTTCTTGGAT-3′) and BMBsp2-*dfrA*-dodo-R (5′-GATAGAGGATGTTAGGTTGGT-3′) (fig. S5, D and E). All primer sequences are listed in data S1.

Whole-genome sequencing of all mutant phages was performed using Illumina MiSeq to verify the specificity of the knockout and exclude potential off-target effects. The sequencing reads were mapped to the reference genome of the wild-type phage using BWA (version 0.7.17) with default parameters (*71*). The alignment results were analyzed using SAMtools (version 1.15) (*72*) to identify any mutations. Integrative Genomics Viewer (*73*) was used for visualization to confirm the precise deletion and assess any off-target modifications (fig. S5, C and F). No off-target mutations were detected, confirming the accuracy of the knockout process.

### Construction of a strain overexpressing the *dfrA53* gene from vB_BtM_BMBsp2 in *B. thuringiensis* YBT-1518

The *B. thuringiensis* vector pHT315*gfp* (*74*), which contains a constitutive promoter pkan and a *gfp* gene under its control, was used to construct a *dfrA53* overexpression vector. We cut out the *gfp* gene using the restriction endonucleases *Xba*I and *Hind*III and ligated it with the *dfrA* gene product, previously digested with the same restriction enzymes, to construct the recombinant plasmid pHT315-*dfrA53*. Subsequently, pHT315-*dfrA53* and pHT315*gfp* were transferred into *B. thuringiensis* strain YBT-1518 by electroporation according to the described method (*75*) to construct the *dfrA53* overexpression strain and the control strain in *B. thuringiensis*.

### RNA extraction and transcription analyses

Total RNA extraction from *E. coli* and *B. thuringiensis* strains was performed by using the TransZol Up Plus RNA Kit (TransGen). Then, the RNA was digested and reverse transcribed to cDNA according to the manufacturer’s protocol (PrimeScript RT reagent Kit with gDNA Eraser; Takara, Dalian, China). The transcription analyses of *dfrA* genes were performed by RT-PCR and qRT-PCR analysis. The expression levels of *dfrA* genes were normalized using the 16*S* ribosomal RNA gene as an internal reference. All primers used for RT-PCR and qRT-PCR are shown in data S1.

### Bacterial growth curves

To determine the impact of *dfrA* gene expression on bacterial growth, we expressed the *dfrA52* gene from *E. coli* phage vB_EcoM_BMB16 in *E. coli* BL21(DE3) using the inducible promoter T7 and the *dfrA53* gene from vB_BtM_BMBsp2 in *B. thuringiensis* YBT1518 using the constitutive promoter pkan, respectively. Overnight cultures of *E. coli* pET21b/BL21, *dfrA52*-pET21b/BL21, and *B. thuringiensis* pHT315*gfp*/YBT1518, pHT315*dfrA53*/YBT1518 were prepared. A 1:100 dilution of these cultures was transferred to fresh LB medium supplemented with ampicillin (100 μg/ml) for *E. coli* and erythromycin (100 μg/ml) for *B. thuringiensis* at 37°C for *E. coli* and 28°C for *B. thuringiensis*, with shaking at 200 rpm until an OD_600_ of 0.4 to 0.5 was reached. Then, a 1:1000 dilution of these cultures was transferred again to fresh medium supplemented with 0.2 mM IPTG and the corresponding antibiotics as described above. To detect growth in the presence of trimethoprim, the corresponding amount of trimethoprim was added to the culture medium. Subsequently, 200 μl of these cultures were carefully deposited into a 100-well honeycomb plate. Bacterial growth was monitored using a Bioscreen C Pro automated growth analyzer at the corresponding temperature, with shaking at 480 rpm and OD_600_ readings were taken at 30-min intervals. These experiments were performed in triplicate.

### Phage reproduction assay

Phage reproduction assay was performed as described by Owen *et al.* (*76*). Specifically, the reporter strains (DH5α for *E. coli* and C15 for *B. thuringiensis*) were cultured overnight and then transferred to fresh LB medium at the corresponding temperature with shaking at 200 rpm, until an OD_600_ of 0.4 to 0.5 was reached. Aliquots (2 ml) of these cultures were then prepared in 15-ml tubes. The phage suspension to be tested was added to achieve a final titer of 100 to 1000 PFU/ml. The infected cultures were then incubated at the corresponding temperature with shaking at 200 rpm. Samples (500 μl) were collected every 30 min and transferred to sterile 1.5-ml centrifuge tubes. Following the incubation times, 500 μl of the cultures were taken and placed in a 1.5-ml sterile centrifuge tube. To stop the infection process, 50 μl of chloroform was added. The mixture was vortexed for 10 s and centrifuged at 20,000*g* for 5 min. The supernatant was collected for 10-fold serial dilutions, and the phage titer was determined using a double-layer overlay plate assay as described above. To measure the phage input at time 0 (T^0^), the same volume of stock phage suspension was added to 2 ml of sterile LB. The phage titer was determined as described above. The fold-reproduction capacity of each phage was calculated as the phage titer of the infected lysate divided by the input phage titer at T^0^.

The phage replication assay in the presence of trimethoprim followed the same protocol as described above. The only modification was the addition of trimethoprim to a final concentration of 4 μg/ml when the phage suspension was added.

To assess the reproduction capacity of Δ*dfrA*_vB_EcoM_BMB16 mutant after coculture with vB_EcoM_BMB16 phage in the presence or absence of trimethoprim after 2 hours, the same method was used as described above. Since vB_EcoM_BMB16 and Δ*dfrA*_vB_EcoM_BMB16 phages could not be distinguished by the double-layer plate method, we used the qRT-PCR method based on absolute quantification to determine the titer of Δ*dfrA*_vB_EcoM_BMB16 phage and calculate its reproduction. Specifically, the total phage titer of the mixture of vB_EcoM_BMB16 and Δ*dfrA*_vB_EcoM_BMB16 phages was determined by using primers encoding the capsid gene (Capsid-qrt-Ec16-F/R). The titer of vB_EcoM_BMB16 phage was then determined using primers encoding the *dfrA* gene (*dfrA52*-qrt-F/R). The titer of Δ*dfrA*_vB_EcoM_BMB16 phage was calculated as the total phage titer minus the titer of vB_EcoM_BMB16 phage. To generate standard curves for absolute quantification, the *dfrA52* and capsid gene fragments were PCR-amplified, purified, and then quantified using a Qubit fluorometer. The purified PCR products were then serially diluted 10-fold over a range of 10^−9^ to 10^−15^ g/μl. qRT-PCR was performed using 2× Universal SYBR Green Fast qPCR Mix (ABclonal) in an ABI QuantStudio 5 system, in a total reaction volume of 10 μl containing 5 μl of SYBR Green Master Mix, 0.25 μl of each primer (final concentration: 0.25 μM), 1 μl of template DNA, and 3.5 μl of nuclease-free water. The thermal cycling conditions were as follows: an initial denaturation at 95°C for 3 min, followed by 40 cycles of 95°C for 10 s, 60°C for 30 s, and 72°C for 30 s, with a final melt curve analysis from 60 to 95°C in 0.05°C/s increments. Negative controls (no-template controls) were included in all runs to ensure the absence of contamination. Standard curves were generated from the 10-fold serial dilutions of purified PCR products, and amplification efficiency was calculated following standard methods as PCR efficiency = 10^–1/slope^ − 1 (*77*). The amplification efficiency ranged from 94 to 97%, with a coefficient of determination (*R*^2^) > 0.99 for both primer sets (fig. S14). Primer specificity was confirmed by gel electrophoresis and melt curve analysis. Primer sequences are provided in data S1. Absolute quantification of vB_EcoM_BMB16 and total phages was performed on the basis of the standard curve.

To determine the reproduction capacity of phage T7 after coculture with vB_EcoM_BMB16 or Δ*dfrA*_vB_EcoM_BMB16 phages in the presence or absence of trimethoprim, the same sample preparation method as described above was used. Since the plaques of phage T7 were larger than those of vB_EcoM_BMB16 and Δ*dfrA*_vB_EcoM_BMB16 phages (fig. S15), and phage T7 in DH5α had stronger reproduction capacity, the titer of T7 can be easily distinguished by its large phage morphology. All experiments were performed in triplicates.

### Quantification of trimethoprim in culture supernatants by HPLC-MS

To determine whether *dfrA52*-mediated resistance involved degradation of trimethoprim, we quantified trimethoprim concentrations in bacterial culture supernatants using HPLC-MS. A trimethoprim calibration curve was generated using standard solutions prepared in LB medium at final concentrations of 0.3125, 0.625, 1.25, 2.5, 5, 10, and 20 μg/ml. These standards were analyzed by HPLC-MS, and the calibration curve was constructed by plotting trimethoprim concentration (*x* axis) against chromatographic peak area (*y* axis), yielding a linear correlation with an *R*^2^ value of 0.997 (fig. S6A).

For sample preparation, *E. coli* BL21(DE3) strains carrying either the empty pET21b(+) vector or the *dfrA52*-expressing plasmid were grown in LB medium supplemented with trimethoprim (4 μg/ml) at 37°C for 6 hours with shaking. Following incubation, cultures were centrifuged at 12,000*g* for 10 min at 4°C, and the resulting supernatants were collected. Fresh LB medium containing trimethoprim (4 μg/ml) was used as a control. All samples were filtered through a 0.22-μm pore size sterile PES filter to remove residual cells and then subjected to HPLC-MS analysis.

HPLC separation was performed on an Agilent 1260 LC system equipped with an Agilent Eclipse Plus C18 column (250 mm by 4.6 mm, 5-μm particle size). The mobile phase consisted of solvent A (0.1% formic acid in water) and solvent B (0.1% formic acid in acetonitrile). The gradient elution program was as follows: 0 to 1 min: 5% B; 1 to 11 min: 5 to 30% B; 11 to 13 min: 30 to 95% B; 13 to 17 min: 95 to 5% B. The flow rate was set at 0.3 ml/min. Detection and quantification were carried out using an Agilent 6540 UHD Accurate-Mass Q-TOF LC/MS system operated in positive electrospray ionization (ESI+) mode. Trimethoprim concentrations in experimental samples were quantified on the basis of the calibration curve.

### Phage-infection dynamics in liquid medium

Overnight cultures were transferred at a 1:100 dilution into LB medium and incubated at the corresponding temperature with shaking at 200 rpm until the OD_600_ reached 0.4 to 0.5. Then, 180-μl of culture was transferred to a 100-well honeycomb plate containing 20 μl of phage lysate to achieve an MOI of 0.1. Phage infections were performed in triplicate, and the OD_600_ values were monitored using a Bioscreen C Pro automated growth analyzer at the corresponding temperature, with shaking at 480 rpm and OD_600_ readings taken every 30 min. For the *dfrA* overexpression strain and the empty control strain, 1:100 of the overnight culture was transferred and cultured until the OD_600_ reached 0.4 to 0.5, and phage infection dynamics were analyzed as described above.

### CFU and PFU determination

Overnight cultures of the bacterial host (*E. coli* or *B. thuringiensis*) were diluted 1:100 and grown to an OD_600_ between 0.4 and 0.6. The cultures were then infected with the specific phage at an MOI of 0.1 and incubated under the following conditions: *E. coli* at 37°C with shaking, *B. thuringiensis* at 28°C with shaking. At specific time points (e.g., 3 and 6 hours postinfection), 200 μl of samples were collected and centrifuged at 6000 rpm for 3 min. Supernatants were used to measure phage titer using the double-layer overlay plate assay as described above. The pellet was resuspended in phosphate-buffered saline (PBS), washed twice with PBS to remove excess of phages, and then resuspended in 200 μl of PBS, serially diluted, and plated onto LB agar plates to quantify bacterial survival. After overnight incubation, the number of colonies was counted to determine the CFU. These experiments were conducted in triplicates.

### Phage adsorption assay

Adsorption assays for vB_EcoM_BMB16, Δ*dfrA*_vB_EcoM_BMB16, vB_BtM_BMBsp2, and Δ*dfrA*_vB_BtM_BMBsp2 were performed following established protocols (*78*). The assay was performed using *E. coli* DH5α as the host for vB_EcoM_BMB16 and Δ*dfrA*_vB_EcoM_BMB16, and *B. thuringiensis* strain C15 as the host for vB_BtM_BMBsp2 and Δ*dfrA*_vB_BtM_BMBsp2. Bacterial cultures were grown in LB medium under optimal conditions—*E. coli* DH5α at 37°C and *B. thuringiensis* strain C15 at 28°C—until reaching an OD_600_ of approximately 1.0. Phages were then added at an MOI of 0.1, and the mixtures were immediately and thoroughly mixed. Adsorption was conducted at room temperature with gentle shaking (60 rpm) to facilitate phage attachment. To monitor phage adsorption, samples were collected at the following time points: For *E. coli* phages (vB_EcoM_BMB16 and Δ*dfrA*_vB_EcoM_BMB16), every 3 min from 0 to 21 min (i.e., 3, 6, 9, 12, 15, 18, and 21 min). For *B. thuringiensis* phages (vB_BtM_BMBsp2 and Δ*dfrA*_vB_BtM_BMBsp2), every 5 min from 0 to 25 min (i.e., 5, 10, 15, 20, and 25 min). At each time point, 50 μl of chloroform was added to lyse bacterial cells and release nonadsorbed phages. The samples were vortexed for 10 s, centrifuged at 12,000 rpm for 5 min, and the supernatant was collected. Phage titers were determined by serial dilution and the double-layer agar method. To control for potential phage inactivation, a parallel control was prepared by incubating phages in LB medium (without bacteria) and processing them identically. The initial phage titer (0 min) from this control was used as a reference to calculate the percentage of adsorbed phages over time.

### One-step growth curve of phages

To determine whether *dfrA* deletion affects the latent period and burst size of phages, we analyzed the one-step growth curves of both the wild phage and the *dfrA* deletion mutant phages. The one-step growth curve was determined using a standard method (*67*). First, an adsorption rate assay was conducted to determine the optimal adsorption time, which was found to be 15 min (fig. S16). On the basis of this result, we proceeded with one-step growth curve experiments. Specifically, phages were incubated with mid-log phase bacteria (OD_600_ = 0.4 ~ 0.6) at an MOI of 0.1 at room temperature for 15 min to allow sufficient adsorption of phage. Cells were then collected by centrifugation at 13,000*g* for 1 min, and unadsorbed phage particles were removed from the supernatant. The precipitated phage-infected cells were resuspended in 50-ml LB culture medium and incubated at the corresponding temperature, with shaking at 200 rpm. To accurately determine the latent period and burst size, samples were collected every 5 min, and the phage titer of each sample was determined using the double-layer agar plate method. The latent period was determined as the time interval between the initial infection and the first notable increase in phage titer. The burst size of the phage was defined as the ratio of the number of phage particles released at the end of the incubation period relative to the number of bacteria initially infected.

### Expression and purification of EcDHFR, DfrA43, and DfrA52

The *E. coli* BL21(DE3) strain harboring the pET21b(+) plasmid encoding *folA*, *dfrA43*, and *dfrA52* was used for protein expression. Bacterial cultures were grown to mid-log phase (OD_600_ ≈ 0.6 to 0.8) and induced by adding 0.5 mM IPTG overnight at 20°C. Cells were harvested by centrifugation at 8000*g* for 10 min at 4°C, and resuspended in 25 ml of lysis buffer [50 mM tris-HCl, 150 mM NaCl, and 10 mM imidazole (pH 7.5)]. Cell lysis was performed on ice using a low-temperature ultrahigh-pressure continuous flow cell disruptor (JN-MiniPro). The lysate was clarified by centrifugation at 10,000*g* for 60 min at 4°C. The supernatant containing the soluble protein fraction was applied to a Ni2^+^-nitrilotriacetate affinity column (Qiagen) preequilibrated with the lysis buffer. Nonspecifically bound proteins were removed by using a wash buffer [50 mM tris-HCl, 150 mM NaCl, and 60 mM imidazole (pH 7.5)]. The target proteins were then eluted with an elution buffer [50 mM Tris-HCl, 150 mM NaCl, and 300 mM imidazole (pH 7.5)]. Protein purity was confirmed by SDS–polyacrylamide gel electrophoresis. The purified fractions were subsequently buffer-exchanged into 50 mM tris-HCl (pH 7.5) using a HiTrap Desalting Column and stored at −80°C until used.

### Kinetic parameters for the hydrolysis of dihydrofolate by the DHFRs (EcDHFR, DfrA43, and DfrA52) under NADPH condition

The enzymatic activity of DHFR was measured by monitoring the oxidation rate of the reduced form of nicotinamide adenine dinucleotide phosphate (NADPH) at room temperature (~25°C) through the decrease in absorbance at 340 nm according to the previously described method (*25*). The reaction was performed in a buffer containing 50 mM tris-HCl, 50 mM KCl, 0.5 mM EDTA, 10 mM β-mercaptoethanol, bovine serum albumin (BSA, 1 mg/ml), 100 μM NADPH, and DHFR (28 to 53 nM) adjusted to pH 7.5. Before initiating the reaction, the enzyme was preincubated with NADPH for 5 min. The reaction was initiated by adding different concentrations of the substrate dihydrofolate, followed by monitoring the absorbance decrease at 340 nm. The kinetic parameters (Michaelis constant and maximum reaction velocity) were determined by nonlinear regression analysis using GraphPad Prism 8 based on data generated from assays conducted with different dihydrofolate concentrations (5 to 100 μM).

### In vitro catalytic activity reducing dihydrofolate to tetrahydrofolate analysis of EcDHFR, DfrA43, and DfrA52 proteins by HPLC-MS

The enzymatic activity of EcDHFR, DfrA43, and DfrA52 was measured in a 100-μl system containing 50 mM tris-HCl (pH 7.5), 50 mM KCl, 0.5 mM EDTA, 10 mM β-mercaptoethanol, BSA (1 mg/ml), 100 μM NADPH, dihydrofolate (200 μM), and DHFR (28 to 53 nM). The reaction mixture was incubated at room temperature for 15 min. The reaction was then quenched by adding 100 μl of acetonitrile, followed by vortexing and centrifugation. The supernatant was analyzed by HPLC-MS.

HPLC separation was performed using an Agilent 1260 LC system equipped with an Agilent Eclipse Plus C18 column (250 mm by 4.6 mm, 5 μm). The mobile phase consisted of solvent A (0.1% formic acid in water) and solvent B (0.1% formic acid in acetonitrile). The gradient profile was as follows: 0 to 2 min: 5 to 20% B; 2 to 4 min: 20 to 95% B; 4 to 5 min: 95% B; 5 to 5.5 min: 95 to 5% B; 5.5 to 15 min: 5% B. The flow rate was set at 0.3 ml/min. LC-MS analysis was conducted using an Agilent Technologies 6540 UHD Accurate-Mass Q-TOF LC/MS system, operated in positive ESI mode. The instrument settings were as follows: capillary voltage: +3.5 kV; nebulizing gas: nitrogen at 0.31 MPa; drying gas: nitrogen at 10 liter/min with a temperature of 320°C; and nebulizer pressure: 45 psi.

### Statistical analysis

Statistical analyses were performed using SPSS version 22. Data were compared between two groups, with detailed sample sizes and statistical methods provided in data S5.

For normally distributed data, Levene’s test was first used to assess the homogeneity of variances. On the basis of outcome, either the independent samples *t* test (for equal variances) or Welch’s *t* test (for unequal variances) was applied. For nonnormally distributed data, the Mann-Whitney *U* test was used.

Statistical significance thresholds were set as follows: *P* > 0.05, not significant (ns); *P < 0.05; **P < 0.01; ***P < 0.001. All statistical results are provided in data S5.

## Supplementary Materials

### The PDF file includes:

Figs. S1 to S16 Tables S1 and S2 Legends for data S1 to S5 Nucleotide sequence of *dfrA52* and *dfrA53* and their encoded amino acid sequence
