# Supplementary material for: Evolutionary selection of trimethoprim-resistant dfrA genes in lytic phages affects phage and host fitness during infection
Source: Sci Adv. 2025 Sep 26;11(39):eadt4817. doi: 10.1126/sciadv.adt4817 (PMC12466917; doi:10.1126/sciadv.adt4817)
Supplement: Supplementary file 1 — Figs. S1 to S16 Tables S1 and S2 Legends for data S1 to S5 Nucleotide sequence of dfrA52 and dfrA53 and their encoded amino acid sequence [file sciadv.adt4817_sm.pdf]

Science Advances
AAAS

Supplementary Materials for

Evolutionary selection of trimethoprim-resistant *dfrA* genes in lytic phages affects phage and host fitness during infection

Kai Wang *et al.*

Corresponding author: Donghai Peng, donghaipeng@mail.hzau.edu.cn

Sci. Adv. **11**, eadt4817 (2025)
DOI: 10.1126/sciadv.adt4817

The PDF file includes:

Figs. S1 to S16
Tables S1 and S2
Legends for data S1 to S5
Nucleotide sequence of *dfrA52* and *dfrA53* and their encoded amino acid sequence

Other Supplementary Material for this manuscript includes the following:

Data S1 to S5

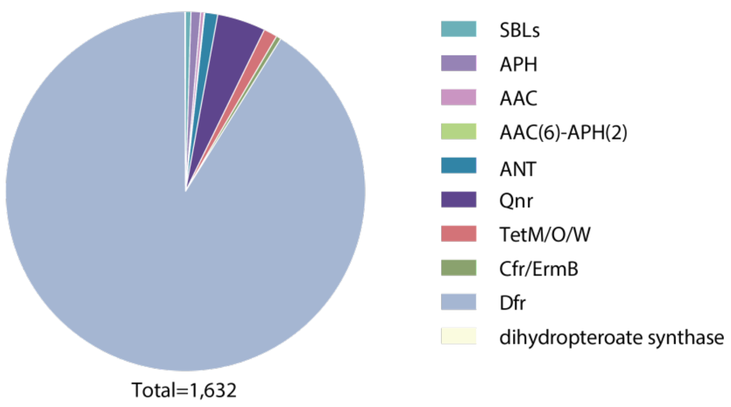

Fig. S1. Analysis of the 1,632 candidate ARGs, concurrently identified by BLASTp, HMMScan, and PCM.

SBLs, serine-β-lactamases; APH, aminoglycoside phosphotransferase; AAC aminoglycoside N-acetyltransferase; AAC(6)-APH(2), aminoglycoside 6'-N-acetyltransferase-aminoglycoside 2'-N-phosphotransferase; ANT, aminoglycoside nucleotidyltransferase; Qnr, quinolone resistance; TetM/O/W, tetracycline resistance protein; Cfr/ErmB, chloramphenicol/florfenicol resistance protein; Dfr, dihydrofolate reductase; dihydrofolate synthase.

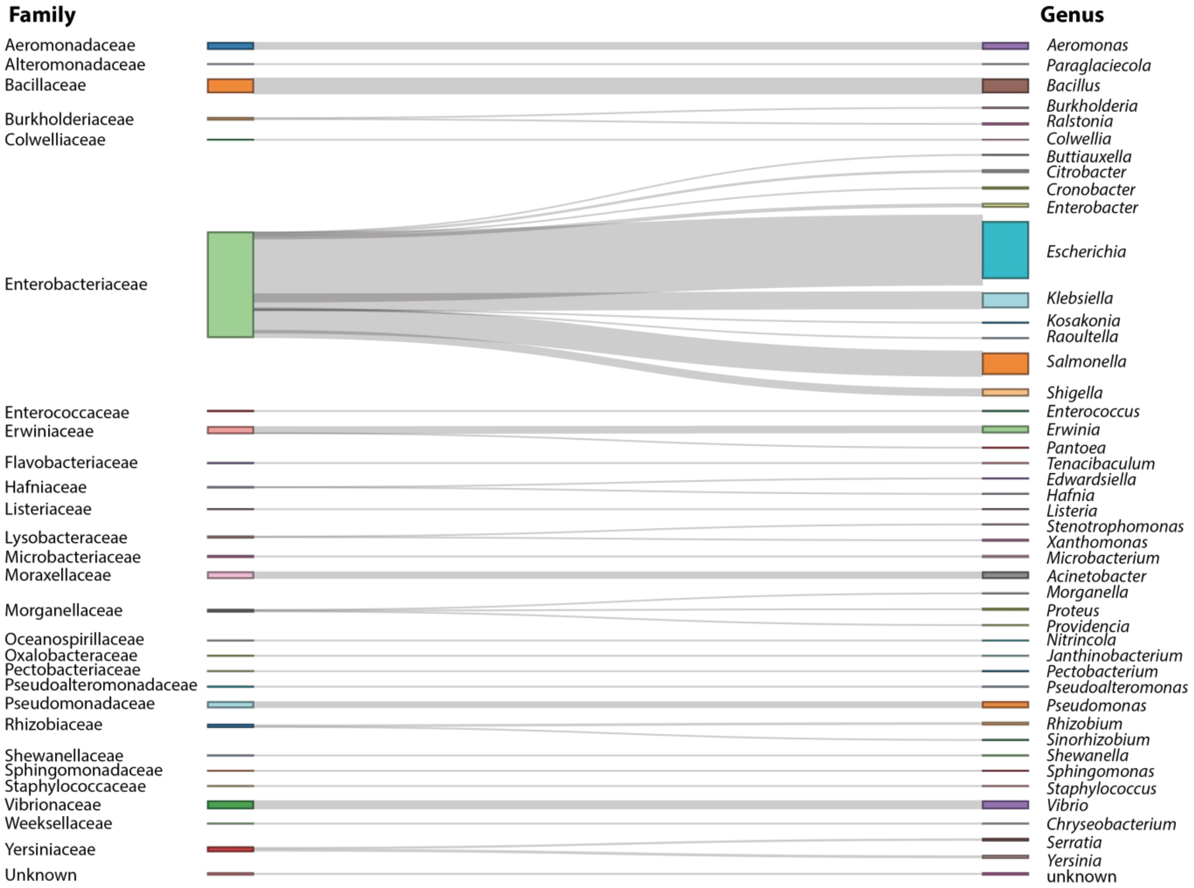

Fig. S2. Statistical analysis of bacterial hosts range of 1,944 lytic phages carrying 1,953 potential *dfrA* genes.

Detailed analysis at the family and genus level was performed on the bacterial hosts of all lytic phages carrying the potential *dfrA* genes, and the results were visualized using a Sankey diagram.

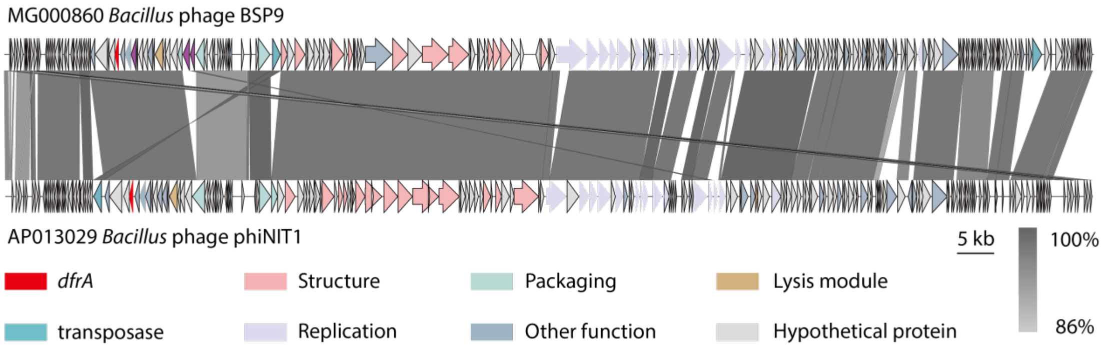

Fig. S3. Whole-genome comparison analysis of *Bacillus* BSP9 and *Bacillus* phiNIT1 phages. The analysis showed that the transposase gene adjacent to potential *dfrA* gene in phiNIT1 phage has undergone horizontal gene transfer, whereas the potential *dfrA* gene itself has not been disseminated. The direction and size of the arrows indicate direction and size of the genes.

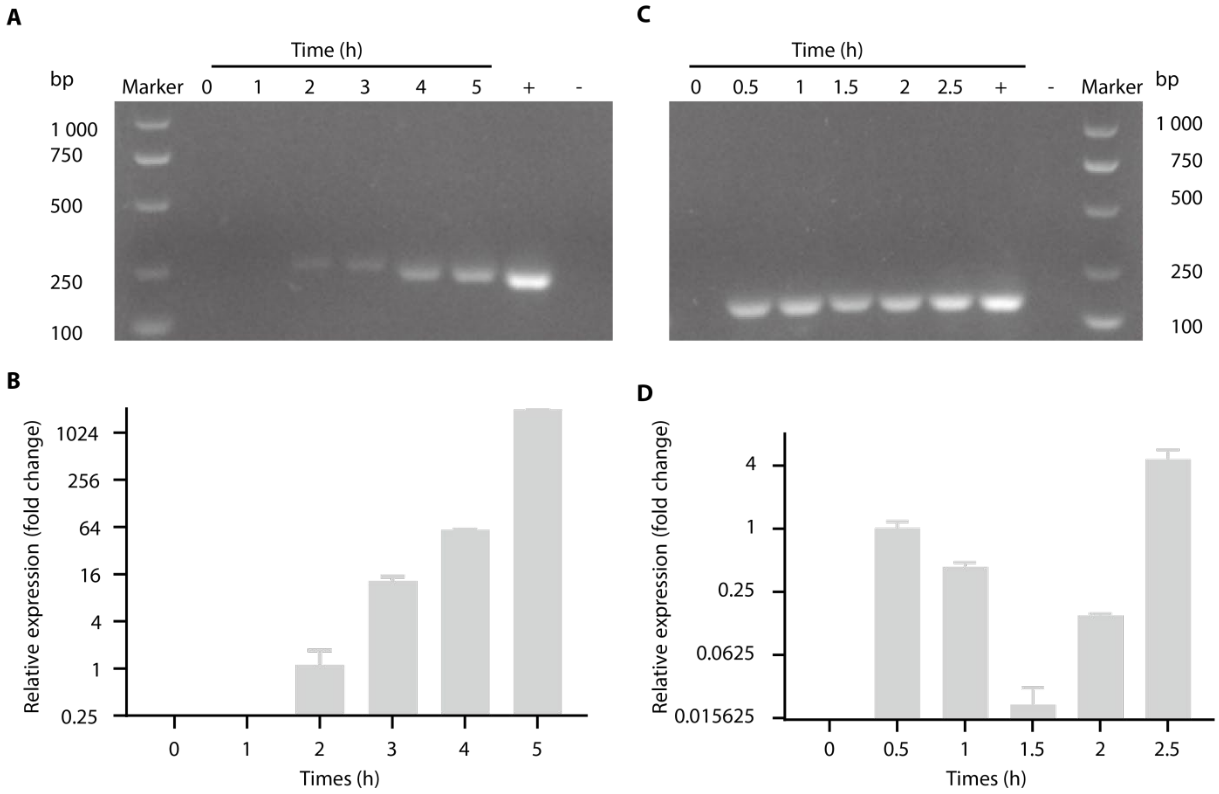

Fig. S4. The *dfrA* genes carried by *E. coli* lytic phage vB_EcoM_BMB16 and *Bacillus* lytic phage vB_BtM_BMBsp2 are transcribed after phage infection.

(A) RT-PCR analysis of *dfrA52* expression after 1 to 5 h of *E. coli* lytic phage vB_EcoM_BMB16 infection. The "+" represents the positive control using the phage genome as a template. **(B)** qRT-PCR analysis of *dfrA52* expression after 1 to 5 h of *E. coli* lytic phage vB_EcoM_BMB16 infection. The expression of *dfrA52* was normalized using the 16S rRNA gene as the reference. The delta-delta Ct method was employed to evaluate changes in *dfrA* expression over time, with the 2 h sample defined as the baseline. Data represent the mean ± standard deviation of n = 3 biological replicates.

(C) RT-PCR analysis of *dfrA53* expression after 0.5 to 3h of *Bacillus* lytic phage vB_BtM_BMBsp2 infection. The "+" represents the positive control using the phage genome as a template. **(D)** qRT-PCR analysis of *dfrA53* expression after 0.5 to 3h of *Bacillus* lytic phage vB_BtM_BMBsp2 infection. The expression of *dfrA53* was normalized using the 16S rRNA gene as the reference. The delta-delta Ct method was employed to evaluate changes in *dfrA* expression over time, with the 0.5 h sample defined as the baseline. Data represent the mean ± standard deviation of n = 3 biological replicates.

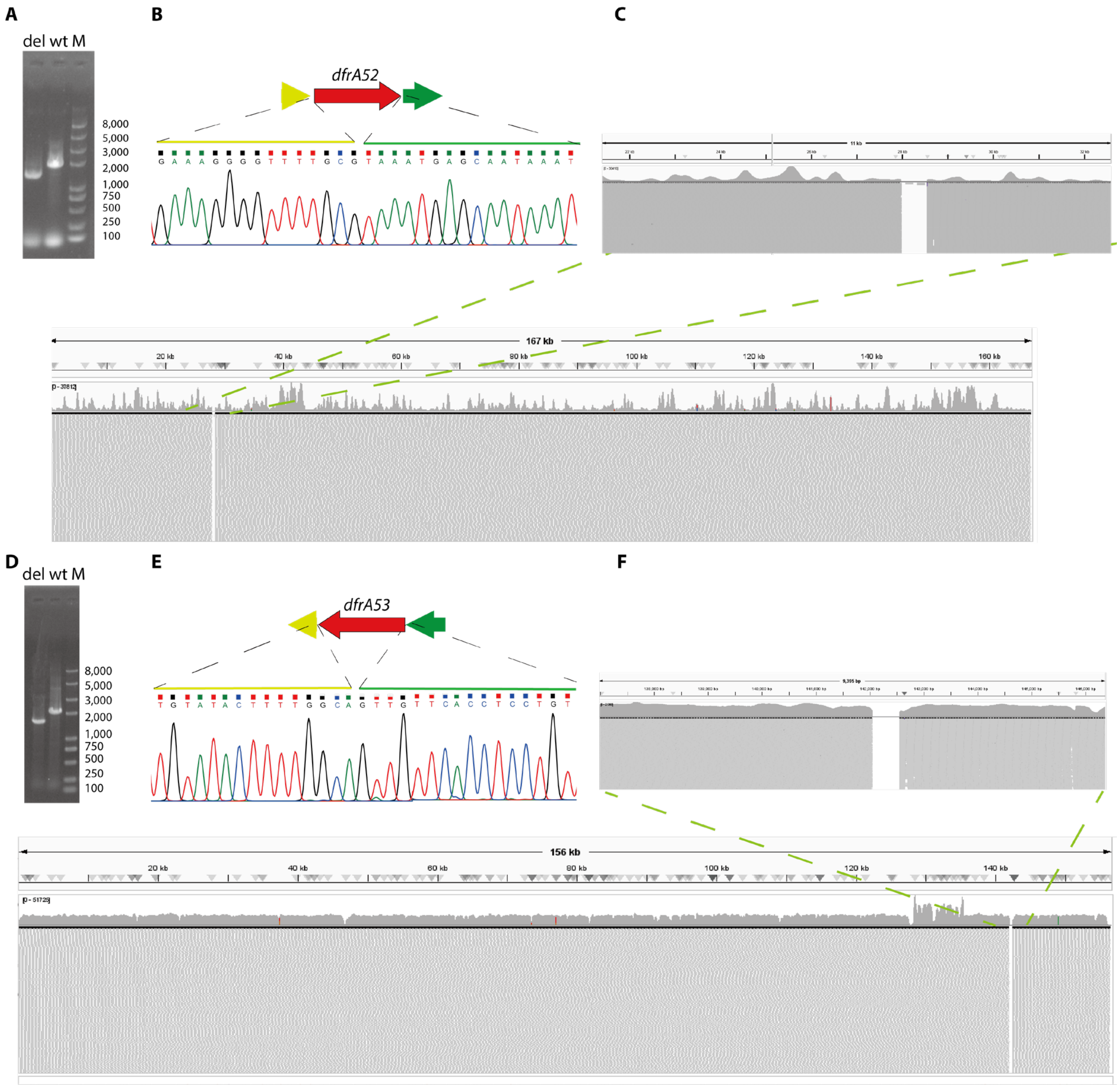

Fig. S5. Validation of *dfrA52* and *dfrA53* knockouts.

(A) PCR verification of *dfrA52* knockout. "del" represents the mutant phage, and "wt" represents the wild-type phage. **(B)** Sanger sequencing chromatogram confirming the deletion of *dfrA52*.

(C) Whole-genome sequencing analysis of the *dfrA52* knockout mutant (Δ*dfrA*_vB_EcoM_BMB16). Reads were mapped to the reference genome, showing that only *dfrA52* was deleted, with no other genetic changes, indicating no off-target effects.

(D) PCR verification of *dfrA53* knockout. "del" represents the mutant phage, and "wt" represents the wild-type phage. **(E)** Sanger sequencing chromatogram confirming the deletion of *dfrA53*. **(F)** Whole-genome sequencing analysis of the *dfrA53* knockout mutant (Δ*dfrA*_vB_BtM_BMBsp2). Reads were mapped to the reference genome, showing that only *dfrA53* was deleted, with no other genetic changes, indicating no off-target effects.

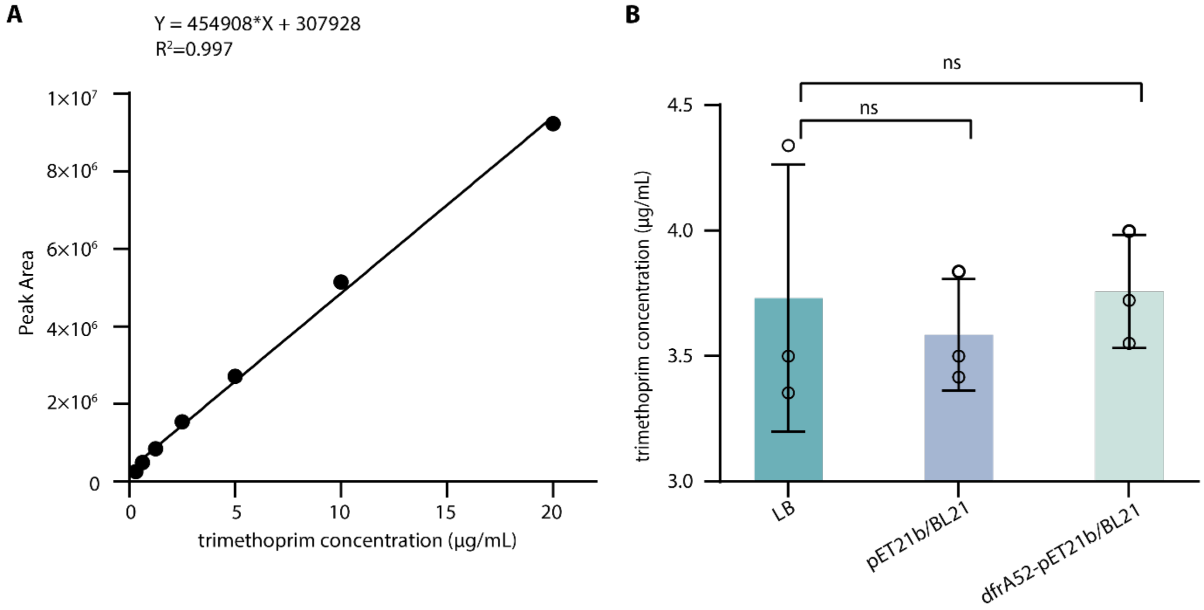

Fig. S6. Quantification of trimethoprim in culture supernatants reveals no antibiotic degradation upon *dfrA52* expression.

(A) Standard calibration curve for trimethoprim generated using HPLC-MS. Trimethoprim concentrations ranging from 0.3125 to 20 µg/mL were prepared in LB medium and analyzed. The chromatographic peak area was plotted against trimethoprim concentration, yielding a linear correlation with $R^2 = 0.997$.

(B) Measurement of trimethoprim concentrations in LB culture supernatants after 6 h of incubation at 37 °C. Three conditions were tested: fresh LB medium containing 4 µg/mL trimethoprim (control), culture supernatant from *E. coli* BL21(DE3) carrying the empty pET21b(+) vector, and supernatant from *E. coli* BL21(DE3) expressing *dfrA52*. No significant change in trimethoprim concentration was observed across the conditions, indicating that *dfrA52*-mediated trimethoprim resistance is not due to antibiotic degradation.

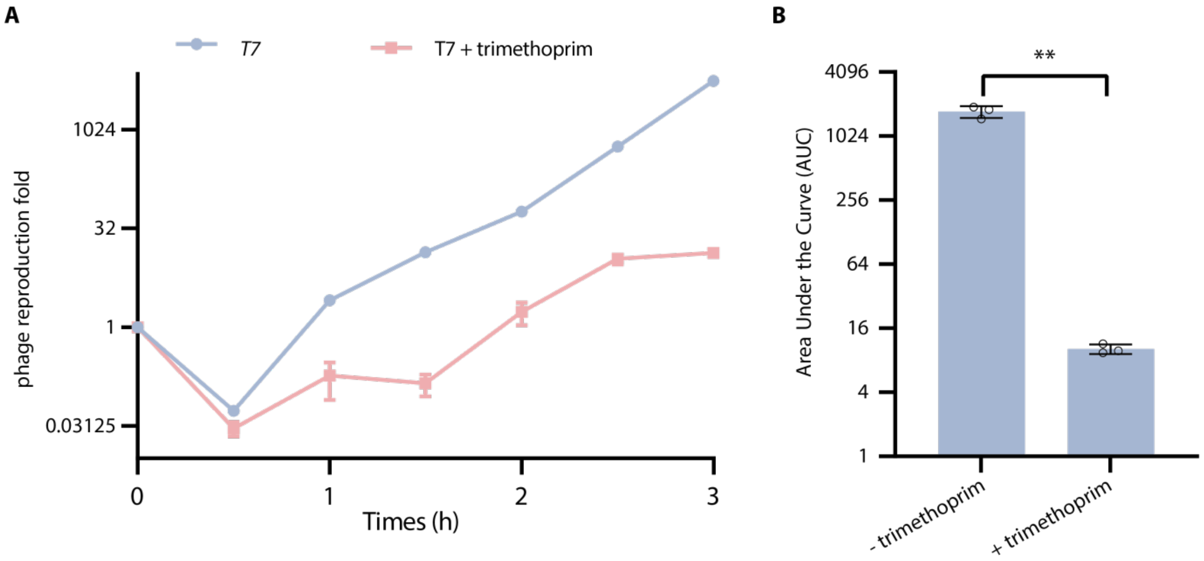

Fig. S7. The reproduction of phage T7 in *E. coli* DH5α was significantly inhibited under trimethoprim pressure.

(A) Reproduction dynamics of phage T7 in *E. coli* DH5α over 3 h under trimethoprim-free conditions and in the presence of 4 μg/mL trimethoprim. Phage replication was measured every 30 min, with the fold change in phage titer calculated as the titer at each time point divided by the titer at 0 h. Data represent the mean ± standard deviation of n = 3 biological replicates.

(B) Quantification of the area under the curve (AUC) from (A), followed by significance analysis. Data represent the mean ± standard deviation of n = 3 biological replicates. Due to a p-value < 0.05 in Levene's test, Welch's t-test was applied for statistical analysis ($p = 0.005672$). Statistical significance thresholds: $p > 0.05$, ns; $p < 0.05$, *; $p < 0.01$, **; $p < 0.001$, ***.

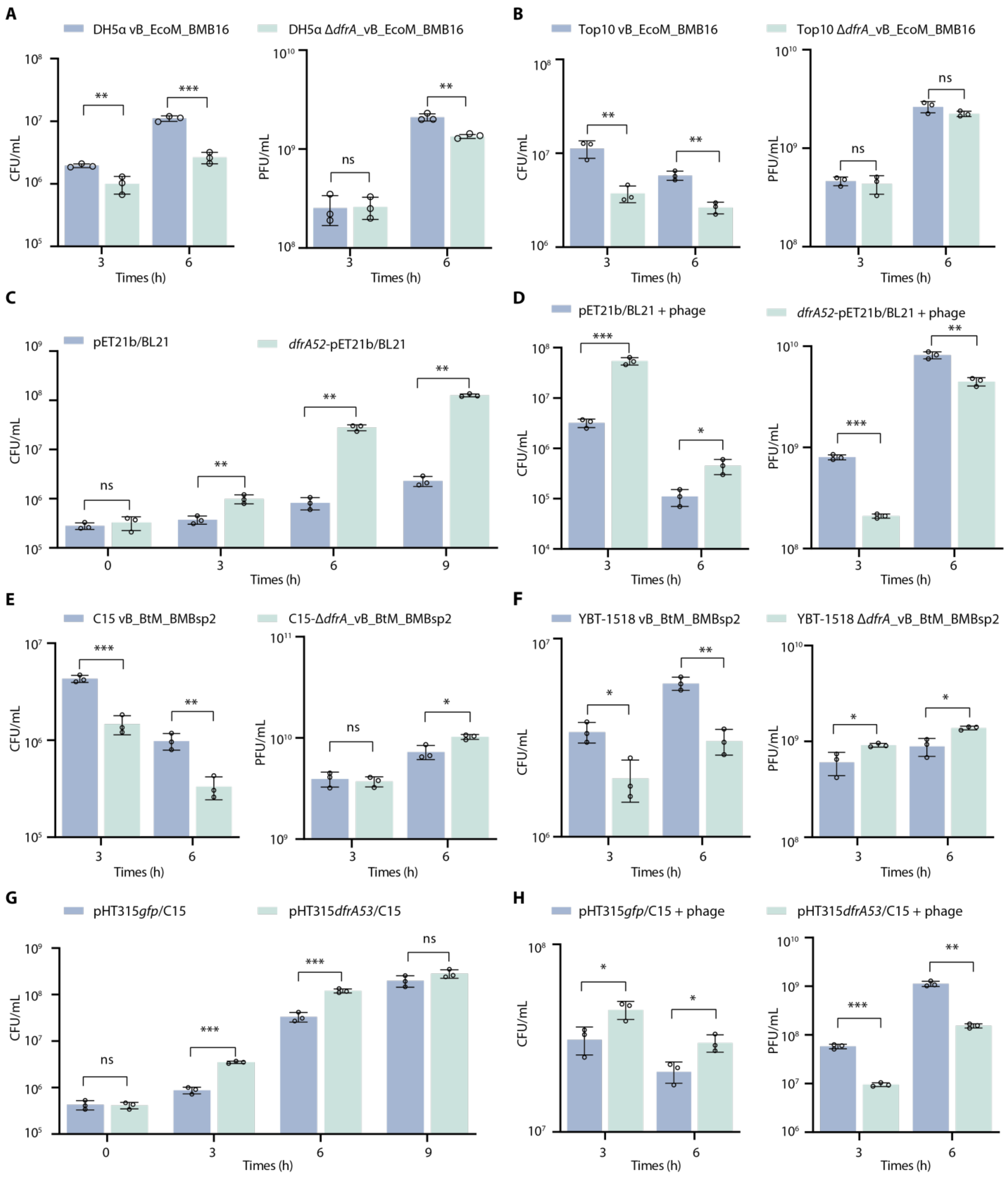

Fig. S8. CFU or PFU measurements across growth curves.

(A) CFU and PFU results at 3 and 6 **h** post-infection of DH5**α** with vB_EcoM_BMB16 and Δ*dfrA*_vB_EcoM_BMB16 phages. Left panel shows CFU data, and right panel shows PFU data. Data represent the mean ± standard deviation of n = 3 biological replicates.

(B) CFU and PFU results at 3 and 6 h post-infection of Top10 with vB_EcoM_BMB16 and Δ*dfrA*_vB_EcoM_BMB16 phages. Left panel shows CFU data, and right panel shows PFU data. Data represent the mean ± standard deviation of n = 3 biological replicates.

(C) CFU data at 0, 3, 6, and 9 h for *E. coli* BL21(DE3) strain expressing the *dfrA52* gene from vB_EcoM_BMB16 phage, and *for* BL21(DE3) strain containing an empty vector as a negative control, after 0.2 mM IPTG induction. Data represent the mean ± standard deviation of n = 3 biological replicates.

(D) CFU and PFU results at 3 and 6 h for *E. coli* BL21(DE3) strain expressing the *dfrA52* gene from vB_EcoM_BMB16 phage, and for BL21(DE3) containing an empty vector as a negative control, both infected with Δ*dfrA*_vB_EcoM_BMB16 phage at an MOI of 0.1 under 0.2 mM IPTG induction. Data represent the mean ± standard deviation of n = 3 biological replicates.

(E) CFU and PFU results at 3 and 6 h post-infection of C15 with vB_BtM_BMBsp2 or Δ*dfrA*_vB_BtM_BMBsp2 phages. Left panel shows CFU data, and right panel shows PFU data. Data represent the mean ± standard deviation of n = 3 biological replicates.

(F) CFU and PFU results at 3 and 6 h post-infection of YBT-1518 with vB_BtM_BMBsp2 or Δ*dfrA*_vB_BtM_BMBsp2 phages. Left panel shows CFU data, and right panel shows PFU data. Data represent the mean ± standard deviation of n = 3 biological replicates.

(G) CFU data at 0, 3, 6, and 9 h for *Bacillus thuringiensis* C15 strain expressing the *dfrA53* gene from vB_BtM_BMBsp2 phage, and for C15 strain containing an empty vector. Data represent the mean ± standard deviation of n = 3 biological replicates.

(H) CFU and PFU results at 3 and 6h for *Bacillus thuringiensis* C15 strain expressing the *dfrA53* or *gfp* genes, both infected with ΔdfrA_vB_BtM_BMBsp2 phage at an MOI of 0.1. Data represent the mean ± standard deviation of n = 3 biological replicates.

Statistical significance thresholds: $p > 0.05$, ns; $p < 0.05$, *; $p < 0.01$, **; $p < 0.001$, ***.

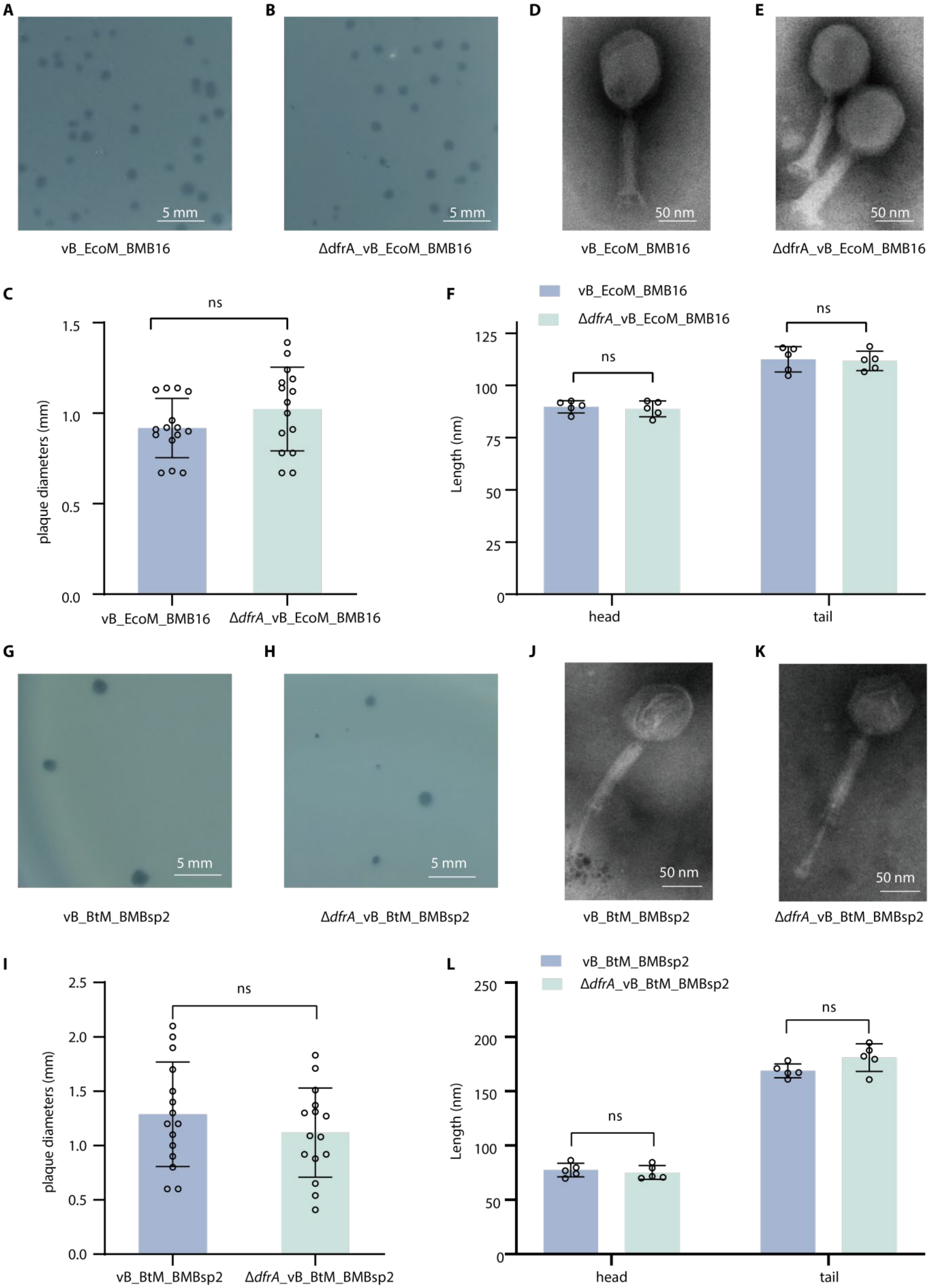

A
5 mm
vB_EcoM_BMB16
B
5 mm
ΔdfrA_vB_EcoM_BMB16
D
50 nm
vB_EcoM_BMB16
E
50 nm
ΔdfrA_vB_EcoM_BMB16
C
ns
plaque diameters (mm)
1.5
1.0
0.5
0.0
vB_EcoM_BMB16
ΔdfrA_vB_EcoM_BMB16
F
vB_EcoM_BMB16
ΔdfrA_vB_EcoM_BMB16
Length (nm)
125
100
75
50
25
0
ns
ns
head
tail
G
5 mm
vB_BtM_BMBsp2
H
5 mm
ΔdfrA_vB_BtM_BMBsp2
J
50 nm
vB_BtM_BMBsp2
K
50 nm
ΔdfrA_vB_BtM_BMBsp2
I
plaque diameters (mm)
2.5
2.0
1.5
1.0
0.5
0.0
ns
vB_BtM_BMBsp2
ΔdfrA_vB_BtM_BMBsp2
L
vB_BtM_BMBsp2
ΔdfrA_vB_BtM_BMBsp2
Length (nm)
250
200
150
100
50
0
ns
ns
head
tail

Fig. S9. The phage plaques and phage morphologies analyses under the transmission electron microscope of *E. coli* phage vB_EcoM_BMB16, *Bacillus* phage vB_BtM_BMBsp2 and their *dfrA* gene deletion mutants.

(A, B) Phage plaques of vB_EcoM_BMB16 **(A)** and Δ*dfrA*_vB_EcoM_BMB16 **(B)** phages infecting *E. coli* DH5α in double-layer agar plates. The scale bar represents 5 mm. **(C)** The mean plaque diameters of vB_EcoM_BMB16 and Δ*dfrA*_vB_EcoM_BMB16 were 0.092 ± 0.016 mm and 0.10 ± 0.023 mm, respectively ($n = 15$, $p = 0.163$), indicating no significant difference.

(G, H) Phage plaques of vB_BtM_BMBsp2 **(G)** and Δ*dfrA*_vB_BtM_BMBsp2 **(H)** phages infecting *B. thuringiensis* C15 in double-layer agar plates. The scale bar represents 5 mm. **(I)** The mean plaque diameters of vB_BtM_BMBsp2 and Δ*dfrA*_vB_BtM_BMBsp2 were 0.13 ± 0.048 mm and 0.11 ± 0.041 mm, respectively ($n = 15$, $p = 0.314$), showing no significant difference.

(D, E, J, K) Transmission electron microscopy image of vB_EcoM_BMB16 **(D)**, Δ*dfrA*_vB_EcoM_BMB16 **(E)**, vB_BtM_BMBsp2 **(J)** and Δ*dfrA*_vB_BtM_BMBsp2 **(K)** phages. The scale bar represents 50 nm. **(F)** The head diameters of vB_EcoM_BMB16 and Δ*dfrA*_vB_EcoM_BMB16 were 89.80 ± 2.93 nm and 88.79 ± 3.77 nm, respectively, while their tail lengths were 112.50 ± 6.07 nm and 111.74 ± 4.68 nm ($n = 5$, $p = 0.649$ for head diameter; $p = 0.830$ for tail length), indicating no significant difference. **(L)** The head diameters of vB_BtM_BMBsp2 and Δ*dfrA*_vB_BtM_BMBsp2 were 77.32 ± 6.27 nm and 75.16 ± 6.41 nm, respectively, while their tail lengths were 168.83 ± 6.37 nm and 180.94 ± 12.75 nm ($n = 5$, $p = 0.605$ for head diameter; $p = 0.094$ for tail length), also showing no significant difference. Statistical significance thresholds: $p > 0.05$, ns; $p < 0.05$, *; $p < 0.01$, **; $p < 0.001$, ***.

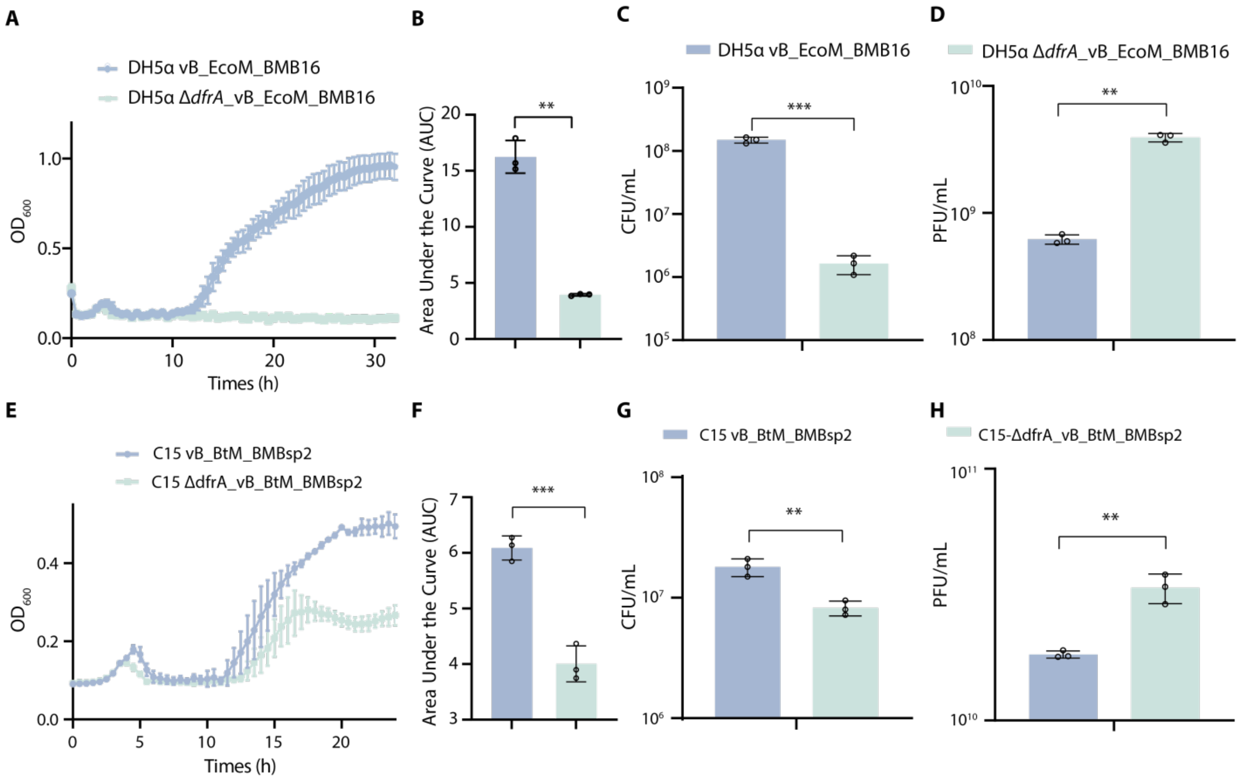

Fig. S10. Growth curves and quantification of CFU and PFU in phage-infected bacterial hosts under low bacterial density conditions.

(A) Growth curves of *E. coli* DH5α strain after infection with vB_EcoM_BMB16 and Δ*dfrA*_vB_EcoM_BMB16 phages at MOI of 0.1 at low bacterial density condition. Data represent the mean ± standard deviation of n = 3 biological replicates. **(B)** Quantification of the area under the curve (AUC) for panel A, followed by significance analysis. Data represent the mean ± standard deviation of n = 3 biological replicates. **(C)** Colony-forming units (CFU) of *E. coli* DH5α cultures at 24 h post-infection. Data represent the mean ± standard deviation of n = 3 biological replicates. **(D)** Plaque-forming units (PFU) of phage titers at 24 h post-infection. Data represent the mean ± standard deviation of n = 3 biological replicates. **(E)** Growth curves of *B. thuringiensis* C15 strain after infection with vB_BtM_BMBsp2 or Δ*dfrA*_vB_BtM_BMBsp2 phages at MOI of 0.1 at low bacterial density condition. Data represent the mean ± standard deviation of n = 3 biological replicates. **(F)** Quantification of the AUC for panel E, followed by significance analysis. Data represent the mean ± standard deviation of n = 3 biological replicates. **(G)** CFU of *B. thuringiensis* C15 cultures at 24 h post-infection. Data represent the mean ± standard deviation of n = 3 biological replicates. **(H)** PFU of phage titers at 24 h post-infection. Data represent the mean ± standard deviation of n = 3 biological replicates. Error bars represent standard deviation, and statistical significance was determined using the appropriate tests (see Data S5). Statistical significance thresholds: $p > 0.05$, ns; $p < 0.05$, *; $p < 0.01$, **; $p < 0.001$, ***.

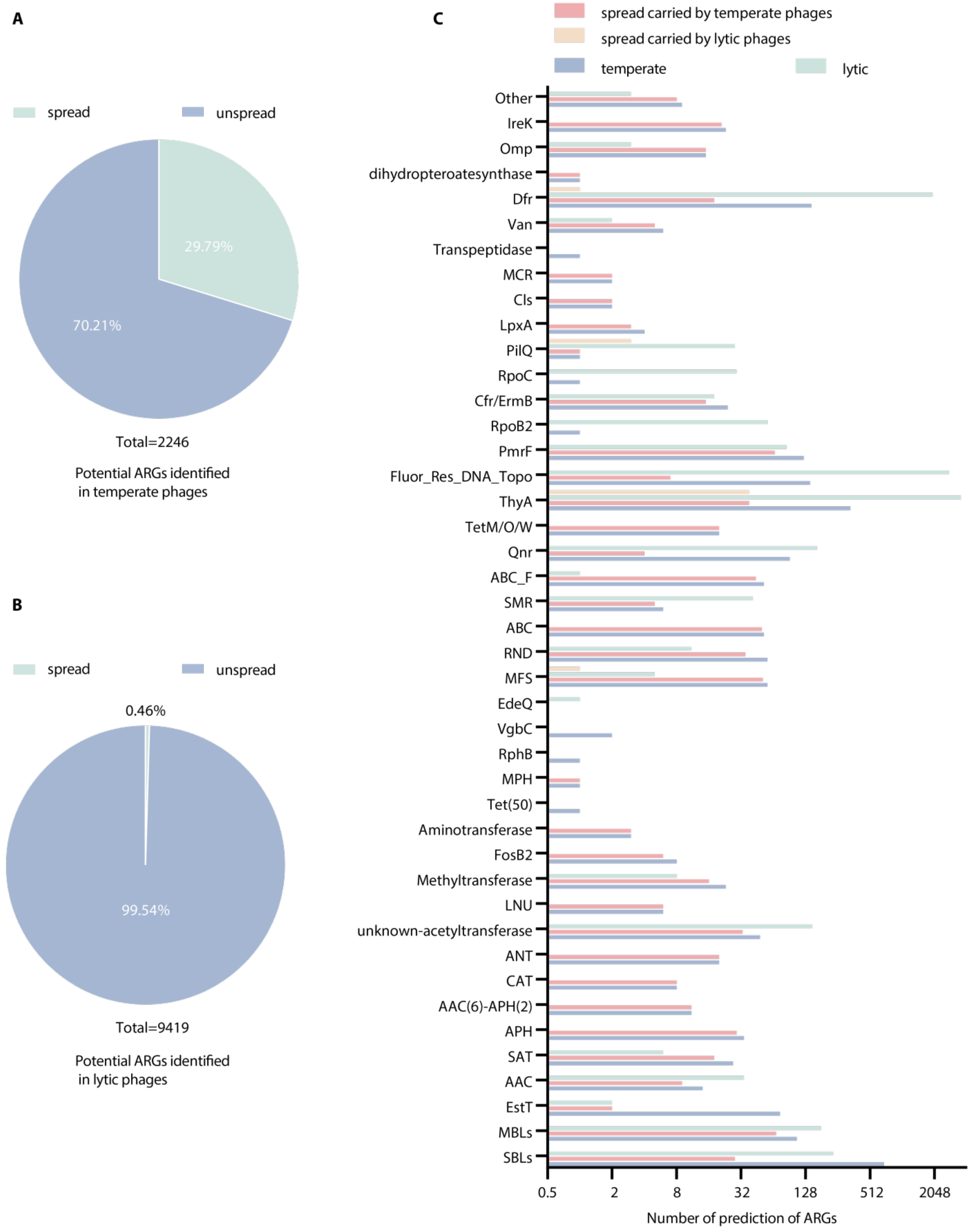
A
spread
unspread
29.79%
70.21%
Total=2246
Potential ARGs identified in temperate phages
B
spread
unspread
0.46%
99.54%
Total=9419
Potential ARGs identified in lytic phages
C
spread carried by temperate phages
spread carried by lytic phages
temperate
lytic
Other
IreK
Omp
dihydropteroatesynthase
Dfr
Van
Transpeptidase
MCR
Cls
LpxA
PilQ
RpoC
Cfr/ErmB
RpoB2
PmrF
Fluor_Res_DNA_Topo
ThyA
TetM/O/W
Qnr
ABC_F
SMR
ABC
RND
MFS
EdeQ
VgbC
RphB
MPH
Tet(50)
Aminotransferase
FosB2
Methyltransferase
LNU
unknown-acetyltransferase
ANT
CAT
AAC(6)-APH(2)
APH
SAT
AAC
EstT
MBLs
SBLs
0.5
2
8
32
128
512
2048
Number of prediction of ARGs

Fig. S11. Lytic phages rarely spread other encoded potential ARGs.

(A) Analysis of the percentage of potential ARGs carried by temperate phages and disseminated within bacterial populations. **(B)** Analysis of the percentage of potential ARGs carried by lytic phages and disseminated within bacterial populations. **(C)** Detailed profile of the dissemination of various types of potential ARGs in bacterial populations, carried by temperate and lytic phages. While pink denotes the transmission of potential ARGs carried by temperate phages to bacteria, orange represents the transmission of potential ARGs carried by lytic phages to bacteria.

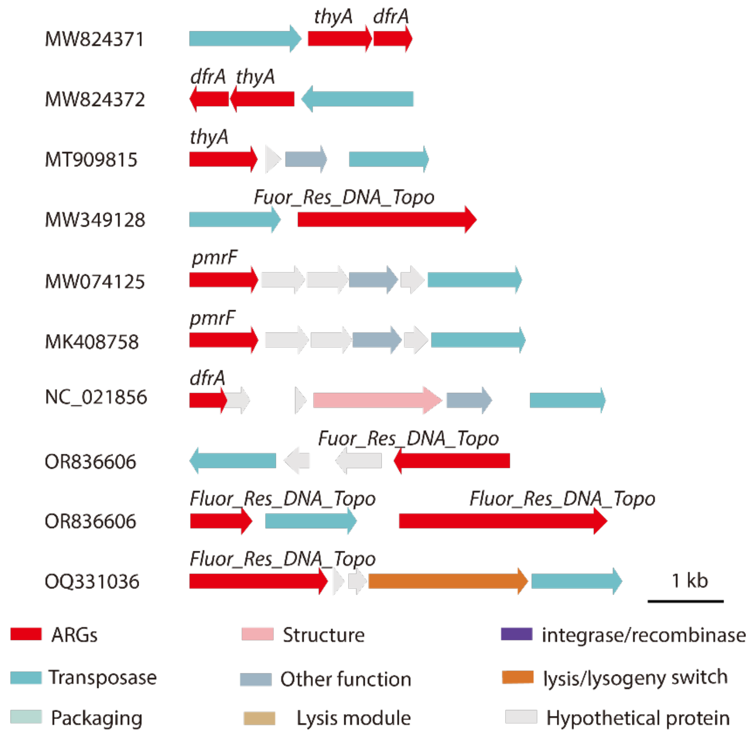

Fig. S12. The profile of potential ARGs adjacent to MGEs in lytic phages.
Genomic organization of lytic phages with mobile elements adjacent to the potential ARGs.

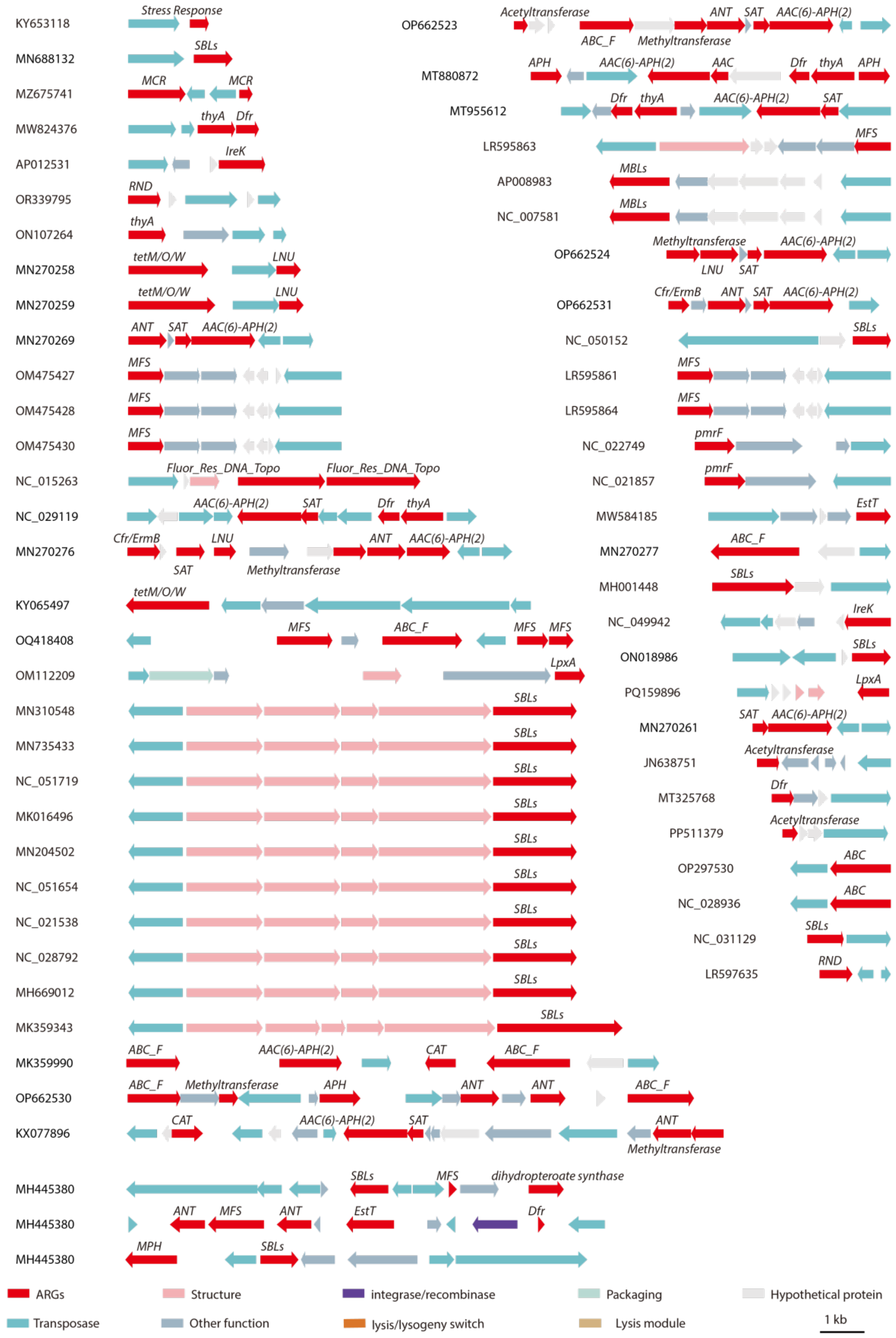

Fig. S13. The profile of potential ARGs adjacent to MGEs in temperate phages.
Genomic organization of temperate phages with mobile elements adjacent to the potential ARGs.

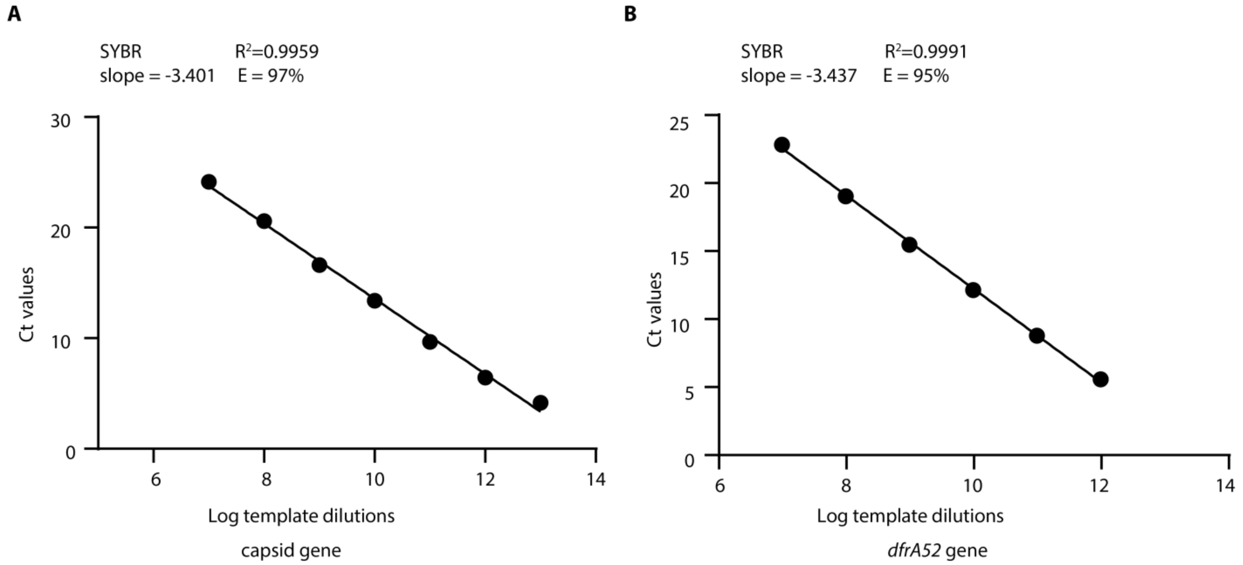

Fig. S14. The qRT-PCR standard curves and amplification efficiency for capsid and *dfrA52* genes of vB_EcoM_BMB16.

Standard curves and amplification efficiency of qRT-PCR for capsid **(A)** and *dfrA52* **(B)** genes. Standard curves were generated using 10-fold serial dilutions of purified PCR products over a concentration range of 10^{-9} g/µL to 10^{-15} g/µL, with Ct values plotted against the logarithm of DNA copy number per milliliter. Amplification efficiency was calculated using the slope of the standard curve, following the standard formula PCR efficiency=$10^{-1/slope} - 1$. The efficiency ranged from 95–97%, with an $R^2 > 0.99$.

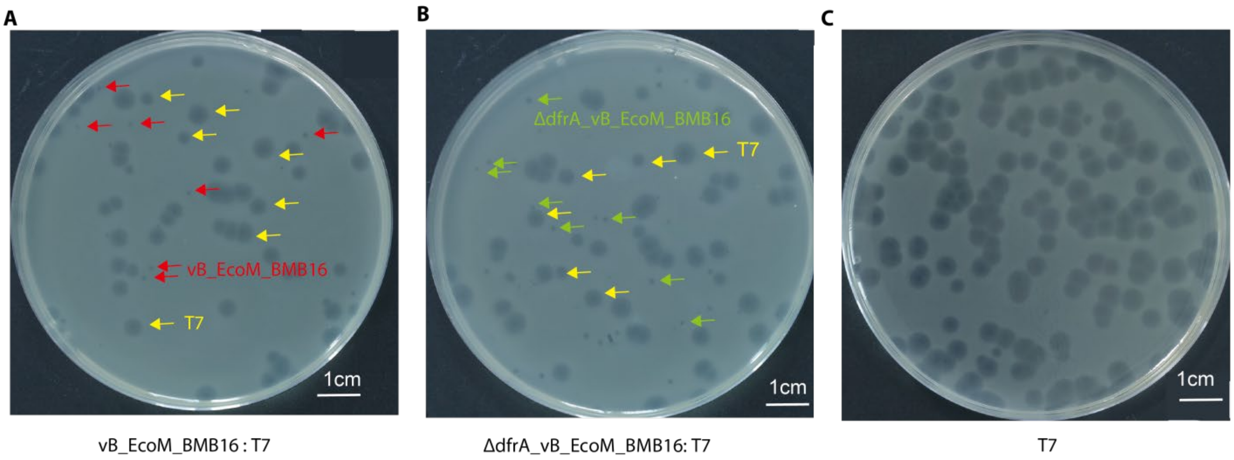

Fig. S15. Phage plaques of vB_EcoM_BMB16 or Δ*dfrA*_vB_EcoM_BMB16 co-infected with T7 or T7 alone infecting *E. coli* DH5α.

(A) Plaque morphology observed in co-infection of vB_EcoM_BMB16 and T7. **(B)** Plaque morphology observed in co-infection of Δ*dfrA*_vB_EcoM_BMB16 and T7. Red arrows indicate vB_EcoM_BMB16 plaques, green arrows indicate Δ*dfrA*_vB_EcoM_BMB16 plaques, and yellow arrows indicate T7 plaques. The scale bar represents 1cm. **(C)** Plaque morphology of T7 infecting *E. coli* DH5α as a reference.

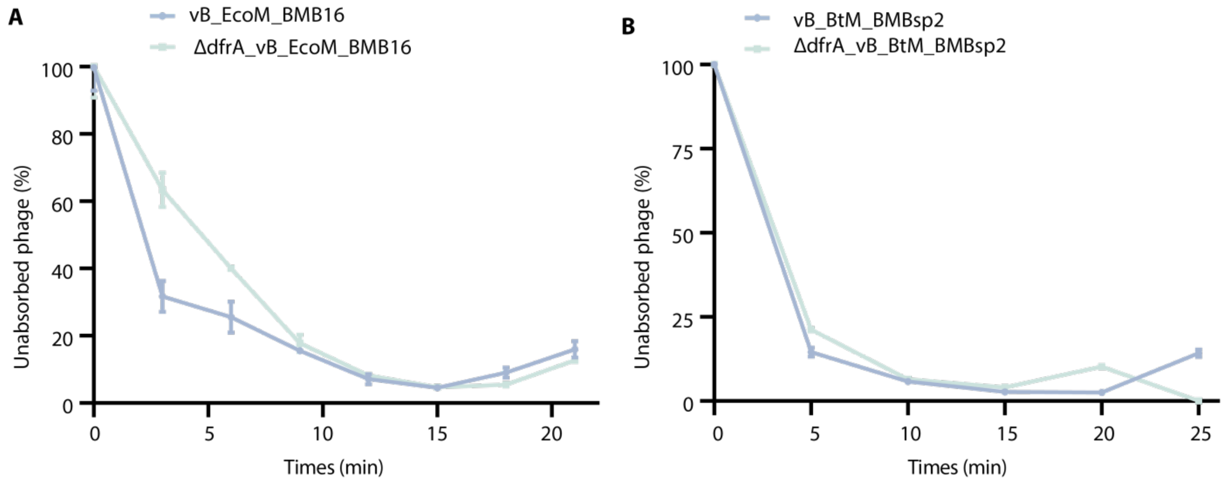

Fig. S16. Adsorption rates of phages vB_EcoM_BMB16, Δ*dfrA*_vB_EcoM_BMB16, vB_BtM_BMBsp2, and Δ*dfrA*_vB_BtM_BMBsp2.

(A) Adsorption rate of phages vB_EcoM_BMB16 and Δ*dfrA*_vB_EcoM_BMB16. The y-axis represents the proportion of un-adsorbed phages in the supernatant. The lowest proportion of un-adsorbed phages is observed at 15 min. Data represent the mean ± standard deviation of n = 3 biological replicates. **(B)** Adsorption rate of phages vB_BtM_BMBsp2 and Δ*dfrA*_vB_BtM_BMBsp2. The y-axis again represents the proportion of un-adsorbed phages in the supernatant, with the lowest proportion of un-adsorbed phages also occurring at 15 min. Data represent the mean ± standard deviation of n = 3 biological replicates.

Table S1. Summary of sensitivity analysis at different identity cutoffs for ARGs Prediction.

Threshold (%)	Total hits	Known ARGs (≥ 90% identity）	Potential ARGs in temperate phages	Potential ARGs in lytic phages	Known ARGs in temperate phages	Known ARGs in lytic phages
90	134	134	134	0	134	0
80	144	134	144	0	134	0
70	157	134	156	1	134	0
60	244	134	207	37	134	0
50	349	134	224	125	134	0
40	1763	134	292	1471	134	0
30	4046	134	685	3361	134	0
E-value thresholds ≤ 1e-5 and query coverage ≥ 60%	9377	134	1234	8143	134	0

Table S2. Trimethoprim susceptibility profile of *E. coli* BL21 cells expressing *dfrA* genes carried by lytic phages.

Strain	The lytic phage carrying *dfrA*-like genes	Virulent genome ID	MIC (mg/L) of trimethoprim
pET21b/BL21			4
folA-pET21b/BL21			16
drfA49-pET21b/BL21			2048
dfrA52-pET21b/BL21	*Escherichia* phage vB_EcoM_BMB16	PV102577.1	256
dfrA53-pET21b/BL21	*Bacillus* phage *vB_BtM_BMBsp2*	OL964058	2048
YP_009304838.1-pET21b/BL21	*Acinetobacter* phage vB_AbaM_phiAbaA1	NC_031280	2048
QZA70902.1-pET21b/BL21	*Erwinia* phage AH06	MZ501268	512
QPX76412.1-pET21b/BL21	*Cronobacter* phage vB_CsaM_SemperBestia	MW021756	1024
QDP45074.1-pET21b/BL21	*Microbacterium* Phage DirtyBubble	MN062714	2048
APU02260.1-pET21b/BL21	*Aeromonas* phage Riv-10	KY290957	2048
ACL78191.1-pET21b/BL21	*Enterobacteria* phage JSE	EU863408	2048
QIG73959.1-pET21b/BL21	*Rhizobium* phage RHph_N34	MN988534	1024
BAN59528.1-pET21b/BL21	*Bacillus* phage phiNIT1 DNA	AP013029	32
ATW69935.1-pET21b/BL21	*Proteus* phage PM135	MG030347	128
QMP82561.1-pET21b/BL21	*Escherichia* phage vB_EcoM_011D4	MT478991	2048
QKE54238.1-pET21b/BL21	*Salmonella* phage vB_SalS_SA001	MN445182	2048

AUV62929.1-pET21b/BL21	*Shigella* phage Sf17	MF327004	2048
AQW88835.1-pET21b/BL21	*Serratia* phage BF	KY630187	2048
QXV79668.1-pET21b/BL21	*Escherichia* phage GreteKellenberger	MZ501072	2048
QTH80474.1-pET21b/BL21	*Pseudomonas* phage pPa_SNUABM_DT01	MW735835	512
AJA41722.1-pET21b/BL21	*Escherichia* phage DT571/2	KM979355	2048
QPI17463.1-pET21b/BL21	*Escherichia* phage T4	MT984581	4

Data S1. (separate file)

Phage isolates, potential ARGs, and experimental resources.
This supplementary section provides detailed data and resources used in the study. It includes the following: List of potential ARGs in all phage isolates; List of all phage isolates and their lifestyle; Strains, plasmids, phages and primers were used in this study; List of sgRNA.

Data S2. (separate file)

Profile of the dissemination of potential ARGs carried by lytic phages and temperate phages in bacteria.

Data S3. (separate file)

Host classification and taxonomic information of all lytic phages carrying potential *dfrA* genes.

Data S4. (separate file)

Accession numbers of potential DfrA proteins encoded by lytic phages and previously reported DfrA proteins were used for phylogenetic analysis.

Data S5. (separate file)
Summary of statistical analyses for all experimental data.

Nucleotide sequence of *dfrA52* and *dfrA53* and their encoded amino acid sequence.

> *dfrA52*

ATGATTAAATTGGTATTCGCTTATTCTCCGACTAAAACAGTCGAAGGCTTTAATGAA

TTAGCATTCGGCTTAGGTGATGGTTTACCATGGGGACGAGTTAAAAAGGACCTTCAG

AATTTTAAAGCTCGTACTGAAGGCACGATTATGATTATGGGTGCTAAAACGTTCCAG

TCATTGCCTACATTACTTCCAGGTCGTAGACATATTGTAGTGTGTGACCTCGAGCGT

GATTATCCTGAAACTAAAGACGGTGATTTAGCACATTTCTATATTACATGGGAGCAG

TACATAACTTACATTTCTGGCGGTTCAGTTCAAATATCAAGCCCTAATGCACCATTC

GAGGCTATGCTTGACCAGAATTCTAATGTAAGTGTAATTGGCGGACCCGCTCTACTA

TATGCTGCATTACCTTATGCAGATGAAGTAGTTGTTTCTCGCATCGTTAAAAGGCAT

CGTGTTAATTCAACGGTTCAATTAGATGCAAGTTTTCTTGATGATATAAGCAAACGT

GAAATGGTTGAAAGTCATTGGTATAAAATAGATGAAGTAACAACCCTTACGGAATC

AGTATATAAATGA

>DfrA52_XPO54507.1

MIKLVFAYSPTKTVEGFNELAFGLGDGLPWGRVKKDLQNFKARTEGTIMIMGAKTFQSL

PTLLPGRRHIVVCDLERDYPETKDGDLAHFYITWEQYITYISGGSVQISSPNAPFEAMLD

QNSNVSVIGGPALLYAALPYADEVVVSRIVKRHRVNSTVQLDASFLDDISKREMVESHW

YKIDEVTTLTESVYK*

>*dfrA53*

ATGGAAATATCATTAATAGCAGCTATCGGAAAGAACAACGAGATTGGGTTAGACAA

TAAGTTATTGTGGCGTTGTAAGGAAGATTTCGACTGGTTTAAGAAGCATACATATAA

CAAACCTGTAGTAATGGGTAGAAAGACATATGAAAGCATCGGTAAACCACTTCCAG

GACGAATCAATATCGTACTGAGTCGAGATGAGAGTTACGATCCACATGAATCTGTAC

TTGTCCTACCTAGTGTGGCCGCAGTCTTTAGTGAACTGAAAAAGTACAGAGAGGTTA

TGATTATCGGTGGTGCAAACGTGTACAAACAGTTTATGCCATTCGCTAACAGATTAT

ACCTAACTGAACTAGATAAAGAGTTTGAAGCAGATAGTTTCTTTCCTACCTTTGTAA

AGGATGATTACCGAGAGTGTTTCGGTGAAGAGGGTACGGAGAAAGTTGGTTTCAAG

TACGAGTTCAAAGTATACCGTAAAAAATTAAGCAAGGAGGGGCAATAA

>DfrA53_UJH95751.1

MEISLIAAIGKNNEIGLDNKLLWRCKEDFDWFKKHTYNKPVVMGRKTYESIGKPLPGRI

NIVLSRDESYDPHESVLVLPSVAAVFSELKKYREVMIIGGANVYKQFMPFANRLYLTEL

DKEFEADSFFPTFVKDDYRECFGEEGTEKVGFKYEFKVYRKKLSKEGQ*
